# Show me your secret(ed) weapons: a multifaceted approach reveals a wide arsenal of type III‐secreted effectors in the cucurbit pathogenic bacterium *Acidovorax citrulli* and novel effectors in the *Acidovorax* genus

**DOI:** 10.1111/mpp.12877

**Published:** 2019-10-23

**Authors:** Irene Jiménez‐Guerrero, Francisco Pérez‐Montaño, Gustavo Mateus Da Silva, Naama Wagner, Dafna Shkedy, Mei Zhao, Lorena Pizarro, Maya Bar, Ron Walcott, Guido Sessa, Tal Pupko, Saul Burdman

**Affiliations:** ^1^ Department of Plant Pathology and Microbiology The Robert H. Smith Faculty of Agriculture, Food and Environment The Hebrew University of Jerusalem Rehovot Israel; ^2^ Department of Microbiology University of Seville Seville Spain; ^3^ The School of Molecular Cell Biology and Biotechnology The George S. Wise Faculty of Life Sciences Tel Aviv University Tel Aviv Israel; ^4^ Department of Plant Pathology University of Georgia Athens GA USA; ^5^ Department of Plant Pathology and Weed Research Agricultural Research Organization The Volcani Center Bet Dagan Israel; ^6^ School of Plant Sciences and Food Security The George S. Wise Faculty of Life Sciences Tel Aviv University Tel Aviv Israel

**Keywords:** *Acidovorax citrulli*, bacterial fruit blotch, effectors, HrpX, machine learning, RNA‐Seq, type III secretion

## Abstract

The cucurbit pathogenic bacterium *Acidovorax citrulli* requires a functional type III secretion system (T3SS) for pathogenicity. In this bacterium, as with *Xanthomonas* and *Ralstonia* spp*.*, an AraC‐type transcriptional regulator, HrpX, regulates expression of genes encoding T3SS components and type III‐secreted effectors (T3Es)*.* The annotation of a sequenced *A. citrulli* strain revealed 11 T3E genes. Assuming that this could be an underestimation, we aimed to uncover the T3E arsenal of the *A. citrulli* model strain, M6. Thorough sequence analysis revealed 51 M6 genes whose products are similar to known T3Es. Furthermore, we combined machine learning and transcriptomics to identify novel T3Es. The machine‐learning approach ranked all *A. citrulli* M6 genes according to their propensity to encode T3Es. RNA‐Seq revealed differential gene expression between wild‐type M6 and a mutant defective in HrpX: 159 and 28 genes showed significantly reduced and increased expression in the mutant relative to wild‐type M6, respectively. Data combined from these approaches led to the identification of seven novel T3E candidates that were further validated using a T3SS‐dependent translocation assay. These T3E genes encode hypothetical proteins that seem to be restricted to plant pathogenic *Acidovorax* species. Transient expression in *Nicotiana benthamiana* revealed that two of these T3Es localize to the cell nucleus and one interacts with the endoplasmic reticulum. This study places *A. citrulli* among the ‘richest’ bacterial pathogens in terms of T3E cargo. It also revealed novel T3Es that appear to be involved in the pathoadaptive evolution of plant pathogenic *Acidovorax* species.

## Introduction

The genus *Acidovorax* contains a variety of species with different lifestyles. While some are well adapted to soil and water environments, others have developed intimate relationships with eukaryotic organisms, including as plant pathogens (Rosenberg *et al.*, 2015). Among the latter, *Acidovorax citrulli* is one of the most important plant pathogenic species (Burdman and Walcott, 2018). This bacterium infects all aerial parts of cucurbit plants, causing bacterial fruit blotch (BFB) disease. The unavailability of effective tools for managing BFB and the disease’s high destructive potential exacerbate the threat BFB poses to cucurbit production (Burdman and Walcott, 2012; Zhao and Walcott, 2018) yet little is known about basic aspects of *A. citrulli*–plant interactions.

On the basis of genetic and biochemical features, *A. citrulli* strains are divided into two main groups: group I strains have been generally isolated from melon and other non‐watermelon cucurbits, whereas group II strains have been mainly isolated from watermelon (Burdman *et al.*, 2005; Walcott *et al.*, 2000, 2004). *Acidovorax citrulli* M6 is a group I strain that was isolated in 2002 from a BFB outbreak in melons (Burdman *et al.*, 2005) and subsequently became a model group I strain for investigation of basic aspects of BFB. Its genome has been sequenced, first by Illumina MiSeq (Eckshtain‐Levi *et al.*, 2016) and more recently by PacBio (Yang *et al.*, 2019), which allowed its complete closure.

As with many Gram‐negative plant and animal pathogenic bacteria, *A. citrulli* relies on a functional type III secretion system (T3SS) to promote disease (Bahar and Burdman, 2010). T3SSs are employed by these pathogens to deliver protein effectors into target eukaryotic cells. Collectively, type III‐secreted effectors (T3Es) promote disease by modulating a variety of cellular functions for the benefit of the pathogen (Block *et al.*, 2008; Büttner, 2016; Galan *et al.*, 2014). In the case of plant pathogenic bacteria, T3Es promote virulence through alteration of the plant cell metabolism and/or suppression of host immune responses (Feng and Zhou, 2012; Macho and Zipfel, 2015). As part of their defence mechanism, plants recognize some effectors by corresponding disease resistance (R) proteins, mostly belonging to the nucleotide‐binding (NB)‐leucine‐rich repeat (LRR) type of immune receptors (NLRs) (Duxbury *et al.*, 2016; Jones and Dangl, 2006). On effector recognition, R proteins elicit a battery of defence responses collectively referred to as effector‐triggered immunity (ETI). ETI is often accompanied by the hypersensitive response (HR), a rapid death of plant cells at the infection site that arrests pathogen spread in the plant tissue (Flor, 1971). Elucidating the arsenal of pathogen effectors and their contribution to virulence is therefore of critical importance for the understanding of basic aspects of pathogenicity and also for translational research in the crop protection field.

Due to the requirement of type III secretion (T3S) for pathogenicity in susceptible plants and HR elicitation in resistant plants, the genes encoding key T3SS regulators and structural components in plant pathogenic bacteria are named *hrp* genes (for HR and pathogenicity) or *hrc* genes, in the case of *hrp* genes that are conserved among different bacterial genera (Bogdanove *et al.*, 1996). On the basis of gene content, operon organization and regulation, *hrp* clusters are divided into class I, which contains the *hrp* clusters of *Pseudomonas syringae* and plant pathogenic bacteria from the *Enterobacteriaceae* family, and class II, which contains the clusters of *Xanthomonas* species, *Ralstonia solanacearum* and plant pathogenic *Acidovorax* spp. (Bahar and Burdman, 2010; Bogdanove *et al.*, 1996; Büttner and Bonas, 2002).

In *Xanthomonas* spp. and *R. solanacearum*, the expression of *hrp*‐, *hrc*‐ and *hrp*‐associated (*hpa*) genes, as well as some T3E genes, is regulated by HrpG and HrpX/HrpB (HrpX in *Xanthomonas* spp. and HrpB in *R. solanacearum*). HrpG belongs to the OmpR family of two‐component system response regulators and controls transcription of *hrpX*/*hrpB* (Büttner and Bonas, 2010; Genin and Denny, 2012; Wengelnik *et al.*, 1996a). *hrpX* and *hrpB* encode AraC‐type transcriptional activators that directly mediate the expression of most *hrp*/*hrc* operons and many T3E genes via binding to DNA motifs that are present in their promoter regions. These DNA motifs are named plant‐inducible promoter (PIP) box (TTCGB‐N15‐TTCGB; B being any nucleotide except adenine) in *Xanthomonas* spp. (Wengelnik and Bonas, 1996) and hrp_II_ box (TTCG‐N16_TTCG) in *R. solanacearum* (Cunnac *et al.*, 2004). Recently, Zhang *et al. *(2018) showed that the *hrpG* and *hrpX*/*hrpB* (thereafter *hrpX*) orthologous genes of the *A. citrulli* group II strain Aac5 are required for pathogenicity. They also showed that HrpG activates expression of *hrpX*, which in turn regulates the expression of a T3E gene belonging to the YopJ family.

Until recently, based on the annotation of the genome of the *A. citrulli* group II strain AAC00‐1, we were aware of 11 genes similar to known T3E genes from other bacteria (Eckshtain‐Levi *et al.*, 2014). Considering the higher numbers of T3Es in several other plant pathogenic bacteria, we hypothesized that this could be an underestimation of the actual number of T3Es in *A. citrulli*. We also hypothesized that *A. citrulli* may carry novel T3Es that were not previously described in other bacteria. Guided by these hypotheses, we carried out a detailed sequence analysis of *A. citrulli* M6 open reading frames (ORFs) to identify genes with similarity to known T3E genes from other bacteria. We also combined machine‐learning (ML) and RNA‐Seq approaches to identify putative, novel *A. citrulli* T3Es. Furthermore, we validated a T3E translocation assay to assess T3S‐dependent translocation of candidate effectors. Combining these approaches allowed identification of seven new T3Es that appear to be unique to plant pathogenic *Acidovorax* species. Subcellular localization of three of these T3Es in *Nicotiana benthamiana* was also determined by *Agrobacterium*‐mediated transient expression.

## Results

### Identification of T3E genes of *A. citrulli* by genome annotation, machine learning and sequence analyses

Sequencing of the genome of the group II *A. citrulli* strain AAC00‐1 (GenBank accession CP000512.1) revealed 11 genes similar to T3E genes of other plant pathogenic bacteria (Eckshtain‐Levi *et al.*, 2014). These genes were present in all tested group II strains. In contrast, all assessed group I strains, including M6, lacked the effector gene *Aave_2708* (gene ID according to the AAC00‐1 annotation), encoding a *Xanthomonas euvesicatoria* XopJ homologue. Group I strains also had disrupted ORFs in genes *Aave_3062* and *Aave_2166*, encoding homologues of *Xanthomonas oryzae* pv. *oryzicola* AvrRxo1 and *X. euvesicatoria* AvrBsT, respectively (Eckshtain‐Levi *et al.*, 2014).

To identify new putative T3Es of *A. citrulli* we applied an ML approach that had been successfully used for identification of new T3E genes of *X. euvesicatoria* (Teper *et al.*, 2016) and *Pantoea agglomerans* (Nissan *et al.*, 2018). Using this algorithm, all ORFs of a bacterial genome are scored according to their propensity to encode T3Es. The scoring is based on a large set of features that are described in the Experimental Procedures section. An initial ML run was applied on the ORFs of strain AAC00‐1. This strain, rather than M6, was used for learning and prediction because at the time this ML was conducted, the AAC00‐1 genome was fully assembled with better annotation. For training, the positive set included 12 AAC00‐1 genes that encoded T3E homologues: the 11 genes described by Eckshtain‐Levi *et al. *(2014) and one additional gene, *Aave_2938*, that is identical to *Aave_2708*. The negative set included genes that showed high sequence similarity to ORFs of nonpathogenic *Escherichia coli*.

The output of the ML run is shown it Table S1 (Supporting Information). For each ORF, we searched for the homologue in *A. citrulli* M6. Among the top predictions from AAC00‐1, many genes did not have homologues in M6. As expected, the 12 positive T3E genes of AAC00‐1 were ranked high in this list (among the 36 highest scoring predictions, with 11 being ranked among the top 15; Table S1). Results from this ML run served, together with RNA‐Seq data, for selection of candidate T3E (CT3E) genes for experimental validation (see below).

In parallel, we performed an extensive homology search, using BlastP, to identify additional putative T3E genes of *A. citrulli* M6. This analysis led to the identification of many additional genes with high similarity to T3E genes from other plant pathogenic bacteria. Table 1 summarizes the arsenal of putative T3E genes of *A. citrulli* M6, based on its genome annotation and sequence similarity analysis. Overall, we found 51 putative T3E genes. Most of these genes also received high scores in the ML search, ranking among the top 100 ORFs (Tables 1 and S1). With that said, ten genes encoding T3E homologues were ranked in very low positions in the ML run (Table 1).

**Table 1:** List of putative T3E genes of *Acidovorax citrulli* M6 based on genome annotation and sequence similarity to known T3E genes from other plant pathogenic bacteria.

Locus_tag M6*	Annotation in M6*	Similarity†	ML1‡	ML2‡	Locus_tag AAC00‐1§	X¶	R¶	P¶
***APS58_0030***	*HP*	Type III effector HopBN1	171	8	*Aave_2531*	+	+	+
*APS58_0167* **	*avrBsT*	Avirulence protein AvrBsT	3	15	*Aave_2166*	+	+	+
*APS58_0178*	*HP*	Type III effector HopF2	Not in ML1	131	−	+	(+)	+
***APS58_0492***	*avrPphE*	Avirulence protein AvrPphE family	14	32	*Aave_3452*	+	+	+
***APS58_0502***	*yopJ*	Type III effector YopP/ AvrRxv family	5	23	*Aave_3462*	+	+	(+)
*APS58_0506*	*HP*	Avirulence protein AvrPphE family	Not in ML1	12	−	+	+	+
*APS58_0542*	*hopD2*	Type III effector HopD2/HopAO1	28	26	*Aave_3502*	+	+	+
*APS58_0658*	*HP*	Type III effector XopN	103	13	*Aave_3621*	+	+	+
*APS58_0664*	*HP*	Type III effector XopQ	86	107	*Aave_3626*	+	+	+
***APS58_0885***	*HP*	Type III effector (*R. solanacearum*)	231	81	*Aave_3847*	(+)	+	−
*APS58_1000*	*HP*	Type III effector protein	814	98	*Aave_3961*	+	+	−
*APS58_1023*	*xopD*	Type III effector XopD	265	38	*Aave_4359*	+	(+)	(+)
***APS58_1209***	*HP*	Type III effector YopP/ AvrRxv family	Not in ML1	3	−	−	+	−
***APS58_1255***	*HP*	Type III effector XopAE	161	18	*Aave_4254*	+	−	−
*APS58_1433*	*HP*	Type III effector HopBD1	367	42	*Aave_4427*	+	−	+
***APS58_1482***	*HP*	Type III effector XopF1	241	28	*Aave_4472*	+	−	(+)
*APS58_1627*	*HP*	Type III effector protein	223	143	*Aave_4606*	−	+	−
***APS58_1634***	*HP*	Type III effector XopR	51	24	*Aave_4612*	+	−	−
***APS58_1657***	*HP*	LRR protein, type III effector PopP	73	110	*Aave_4631*	+	+	−
***APS58_1658***	*HP*	LRR protein, outer protein XopAC	43	52	*Aave_4632*	+	(+)	(+)
*APS58_1676*	*HP*	Type III effector protein	Not in ML1	25	−	+	+	(+)
***APS58_1760***	*T3E protein*	Type III effector protein	8	16	*Aave_4728*	+	+	+
*APS58_1921*	*avrPph3*	Cysteine protease avirulence protein YopT/AvrPphB	61	4	*Aave_0085*	+	+	+
*APS58_1966*	*xopJ*	Type III effector XopJ	Not in ML1	9	−	+	+	+
*APS58_2045*	*avrRpt*2	Cysteine protease avirulence protein AvrRpt2	156	14	*Aave_0201*	−	−	+
*APS58_2122*	*xopAG*	Type III effector HopG1/AvrGf1/XopAG	6	10	*Aave_0277*	+	+	+
***APS58_2156***	*HP*	Type III effector XopC2	951	100	*Aave_0310*	+	+	−
***APS58_2228***	*HP*	Type III effector SspH1 family	Not in ML1	45	−	(+)	(+)	−
***APS58_2229***	*putative T3E, E3 ligase domain*	Type III effector SspH1 family	Not in ML1	50	−	(+)	(+)	−
***APS58_2287***	*HP*	Type III effector XopK	235	11	*Aave_0433*	+	−	+
***APS58_2313***	*LRR ribonuclease inhibitor*	LRR type III effector protein (GALA5)	24	71	*Aave_0458*	−	+	−
***APS58_2345***	*HP*	Type III effector XopP	1161	17	*Aave_0588*	+	+	−
*APS58_2589*	*HP*	Type III effector YopP/ AvrRxv family	32	5	*Aave_0889*	+	+	(+)
*APS58_2767*	*HP*	Type III effector protein	Not in ML1	64	−	+	+	−
***APS58_2799***	*HP*	Putative AWR type III effector protein	33	34	*Aave_1090*	−	+	−
*APS58_3109*	*HP*	Avirulence protein AvrXv3	40	36	*Aave_1373*	+	+	+
*APS58_3252*	*HP*	Outer protein XopAC	56	118	*Aave_1508*	+	+	−
***APS58_3261***	*HP*	Type III effector HopBF1	37	60	*Aave_1520*	−	(+)	+
***APS58_3289***	*hopW1‐1*	Type III effector HopW1‐1/HopPmaA	15	19	*Aave_1548*	+	+	+
*APS58_3303*	*HP*	Type III effector XopE2	Not in ML1	1	−	+	+	+
***APS58_3344***	*HP*	Type III effector XopAI	27	93	*Aave_1647*	+	−	(+)
*APS58_3751*	*mltB_2*	Lytic murein transglycosylase, type III effector HopAJ2	36	285	*Aave_3237*	+	+	+
*APS58_3909*	*HP*	Type III effector XopV	193	21	*Aave_3085*	+	+	−
*APS58_3930*	*HP*	Type III effector AvrRxo1‐ORF2	655	31	*Aave_3063*	+	−	−
*APS58_3931 * **	*–*	Type III effector AvrRxo1	13	−	*Aave_3062*	+	−	−
*APS58_3943*	*HP*	Type III effector AvrPphF/HopF2	Not in ML1	37	−	−	+	+
*APS58_4070*	*HP*	Type III effector HopH1	7	6	*Aave_2876*	+	+	+
*APS58_4101*	*HP*	Type III effector, lipase domain	22	22	*Aave_2844*	+	+	−
*APS58_4112*	*avrBs1*	Avirulence protein AvrBs1/AvrA1	4	7	*Aave_2173*	+	−	+
*APS58_4113*	*HP*	Avirulence protein AvrBs1/AvrA1	16	2	*Aave_2174*	+	−	+
***APS58_4317***	*HP*	Type III effector HopD1	34	33	*Aave_2802*	+	+	−

* Locus_tag and annotation according to GenBank accession CP029373.1 (Yang *et al.*, 2019). Bold font indicates genes that were found to be significantly regulated by HrpX based on RNA‐Seq results (Table S2). *HP*, hypothetical protein.

† Similarity based on BlastP analysis of the gene product.

‡ Ranking of the genes in ML runs 1 and 2. ML1 and ML2 were done with ORFs of *A. citrulli* AAC00‐1 (GenBank accession CP000512.1) and *A. citrulli* M6 (GenBank accession CP029373.1), respectively. In column ML1, ‘not in ML1’ means that this M6 gene was not detected in ML1 because it has no homologous gene in strain AAC00‐1.

§ Corresponding locus_tag in *A. citrulli* AAC00‐1. Underlined genes are T3E genes that were known prior to this study, based on the annotation of the *A. citrulli* group II strain AAC00‐1 (Eckshtain‐Levi *et al.*, 2014), in addition to gene *Aave_2708*, which is not present in strain M6.

¶ Similarity to gene products of *Xanthomonas* spp. (X), *Ralstonia* spp. (R) and *Pseudomonas syringae* group (P). + indicates significant similarity to at least one gene product; (+) indicates significant similarity to hits with relatively low query coverage (below 60%); – indicates that no significant hits were detected.

** These genes are probably non‐functional in strain M6 and in all group I strains assessed so far (Eckshtain‐Levi *et al.*, 2014).

Most predicted T3E genes shared levels of similarity with T3E genes of *Xanthomonas* spp. and *R. solanacearum* (44 and 40 genes, respectively; Table 1). A smaller number of genes, 31, shared similarity with T3E genes of *P. syringae* strains. We also assessed the occurrence of these T3Es in other plant pathogenic *Acidovorax* species. Except for the HopBD1 homologue APS58_1433 that could be detected only in *A. citrulli* strains, the other predicted T3Es occur in other pathogenic *Acidovorax* species, with some of them being widely distributed (Table S2).

Interestingly, of the 51 putative T3E genes of *A. citrulli* M6, ten could not be detected in the group II strain AAC00‐1 (Table 1). Besides M6 and AAC00‐1, the NCBI database includes draft genomes of one additional group II strain, KAAC17055, and four group I strains, pslb65, tw6, DSM 17060 and ZJU1106. BlastN analyses revealed that these ten genes are also absent in strain KAAC17055, but are present in most of the group I strains. The only exceptions were *APS58_0506*, which was not detected in strains tw6 and DSM 17060, *APS58_1209*, which was not detected in tw6, and *APS58_2767*, which was not detected in DSM 17060. The inability to detect these T3E genes in the genomes of tw6 and DSM 17060 could reflect true absence in these strains but also could be due to the draft nature of these genomes. In any case, these results strongly suggest that the ten M6 T3E genes that are absent in the sequenced group II strains could be specific to group I strains of *A. citrulli*. This assumption should be verified on a larger collection of strains.

### HrpX regulates expression of T3SS components and T3E genes in *A. citrulli* M6

**Figure 1:**
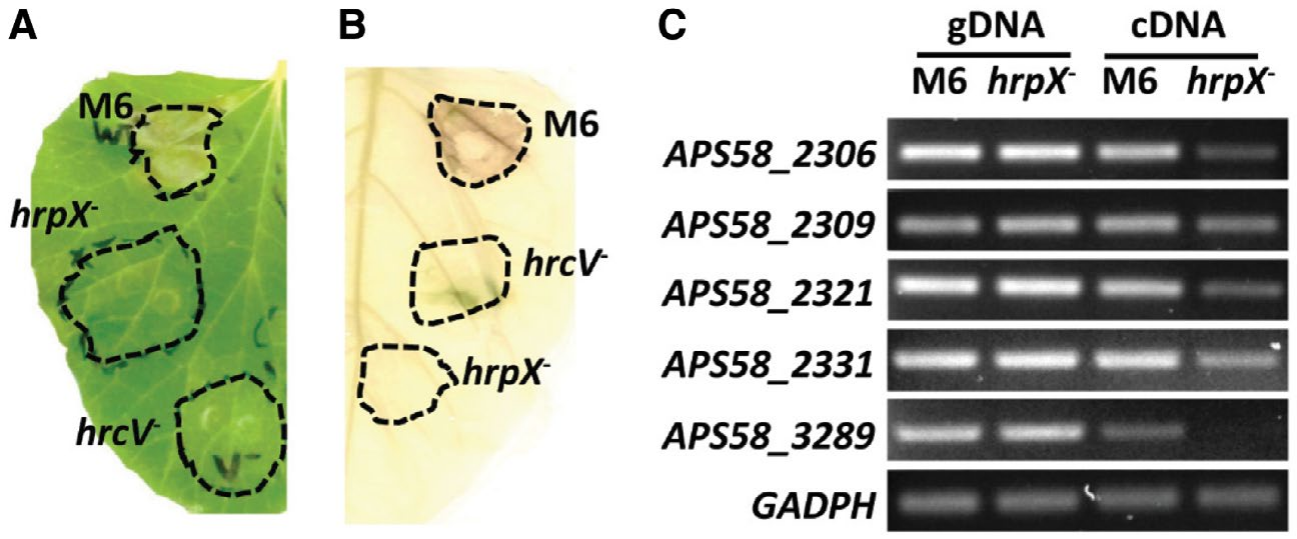
HrpX is required for pathogenicity and regulates expression of T3S and T3E genes in *Acidovorax citrulli* M6. (A) Disease lesions produced in a melon leaf inoculated with wild‐type M6, but not with mutant strains defective in *hrpX* or *hrcV* (encoding a core component of the T3SS) genes. The picture was taken at 3 days after inoculation (dai). (B) Cell death observed in a pepper leaf following inoculation with wild‐type M6, but not with *hrpX* and *hrcV* mutants. The picture was taken at 4 dai. In (A) and (B), leaves were syringe‐infiltrated with a bacterial suspension of 10^8^ cfu/mL. (C) Qualitative assessment of differential gene expression between wild‐type M6 and the M6 *hrpX* mutant after 72 h of growth in XVM2 minimal medium at 28 °C. gDNA, genomic DNA. cDNA, reverse‐trancriptase (RT)‐PCR of RNA extracts. Genes: *hrcV* (*APS58_2306*), *hrcT* (*APS58_2309*), *hrcJ* (*APS58_2321*) and *hrcC* (*APS58_2331*), encoding core T3SS components; *APS58_3289*, encoding a T3E similar to *Pseudomonas syringae hopW1‐1*; *GAPDH*, glyceraldehyde‐3‐phosphate dehydrogenase (*APS58_1610*; control).

In *Xanthomonas* spp. and *R. solanacearum*, the transcriptional regulator HrpX (HrpB in *R. solanacearum*) plays a key role in regulation of *hrp* and T3E genes. We hypothesized that this is also the case in *A. citrulli* M6. To assess this hypothesis, we first generated an *A. citrulli* M6 strain mutated in *APS58_2298,* the *hrpX* orthologous gene. This mutant lost the abilities to cause disease in melon (Fig. 1A) and induce HR in pepper leaves (Fig. 1B). A similar loss of pathogenicity was observed for a mutant defective in the *hrpG* homologue gene *APS58_2299* (Fig. S1). Complementation of *hrpX* and *hrpG* mutations restored pathogenicity, although necrotic symptoms induced by the complemented strains were less severe than those induced by the wild‐type strain (Fig. S1).

Furthermore, we used reverse transcription‐PCR (RT‐PCR) to compare expression of four genes encoding T3SS components and one T3E gene (*APS58_3289*, a *P. syringae hopW1‐1* homologue) between the *hrpX* mutant and wild‐type M6 following growth in XVM2 medium. This medium was optimized for expression of T3S genes in *X. euvesicatoria*, as it simulates, to some extent, the plant apoplast environment (Wengelnik *et al.*, 1996b). After 72 h of growth, expression of the tested genes was reduced in the *hrpX* mutant relative to wild‐type M6 (Fig. 1C).

### Elucidation of the *A. citrulli* HrpX regulon by RNA‐Seq

**Figure 2:**
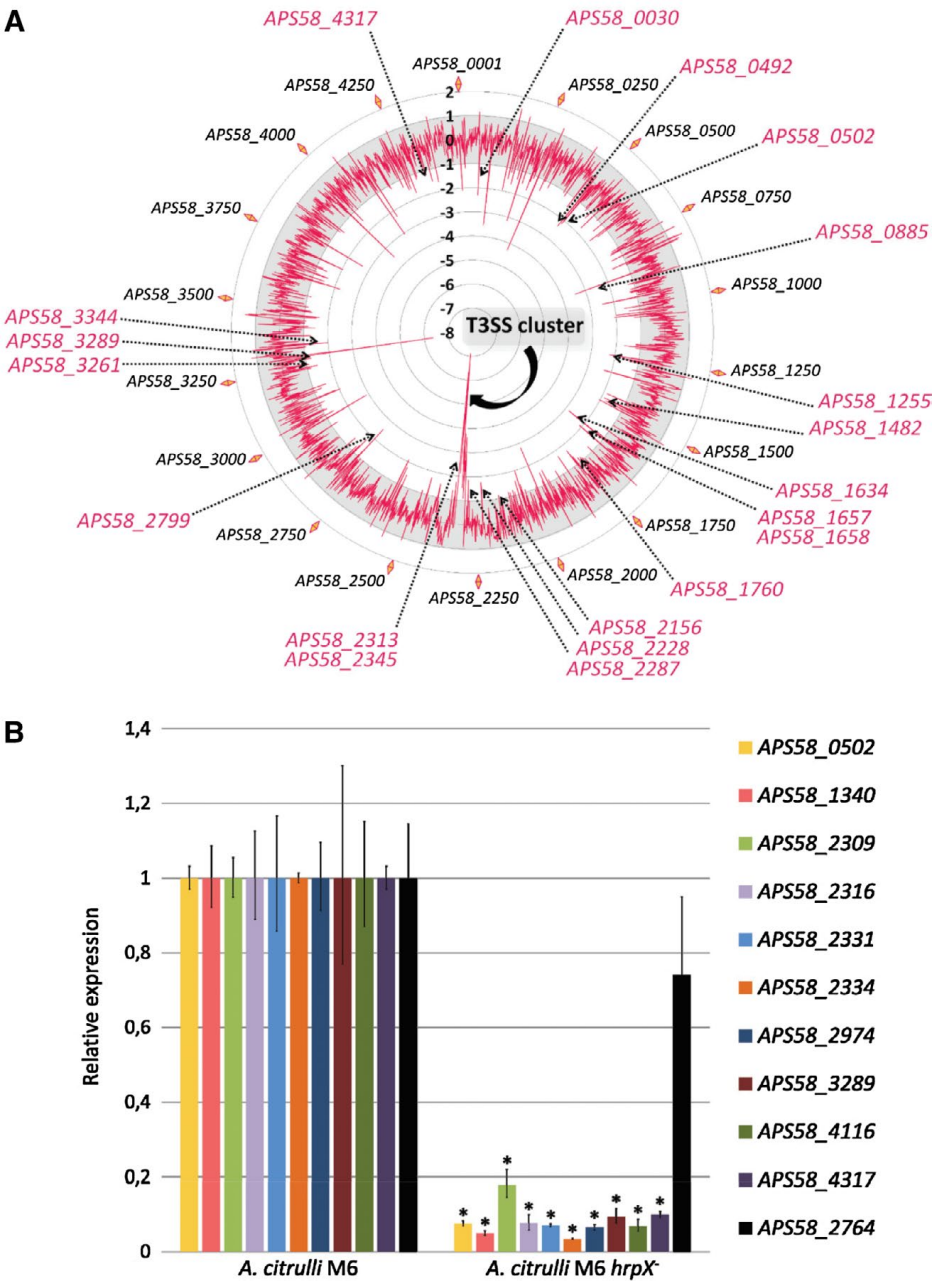
Comparative transcriptomics analysis between *Acidovorax citrulli* M6 and the *hrpX* mutant. (A) Relative gene expression profile as assessed by RNA‐Seq of cells grown for 72 h at 28 °C in XVM2 medium. The *A. citrulli* M6 genome map is represented in the external circle. The internal red line shows differential gene expression between the strains. Genes within the grey zone: no significant differences. The −8 to 2 scale indicates relative expression of the mutant compared with the wild‐type. Genes with significantly reduced or increased expression in the mutant are in the inner and outer regions relative to the grey zone, respectively. Arrows indicate the Hrp‐T3SS cluster as well as genes with homology to known T3Es. (B) Relative expression of selected genes by qRT‐PCR following bacterial growth under identical conditions to the RNA‐Seq experiment (three biological replicates per strain). Asterisks indicate significant differences between wild‐type and *hrpX* mutant at α = 5% by the Mann–Whitney nonparametric test. All tested genes except *APS58_2764* showed significantly reduced expression in the mutant relative to strain M6 in RNA‐Seq.

Based on RT‐PCR results, we carried out RNA‐Seq to compare gene expression between wild‐type M6 and the *hrpX* mutant after 72 h of growth in XVM2 medium. This approach revealed 187 genes showing significant differential expression (significant fold‐change of ±2; *P* < 0.05) between the strains (Fig. 2A). Of these, 159 genes had significantly reduced expression in the *hrpX* mutant relative to wild‐type M6, while 28 genes showed the opposite pattern (Table S3A,B). RNA‐Seq results were validated by qPCR experiments with a set of selected genes (Fig. 2B).

**Figure 3:**
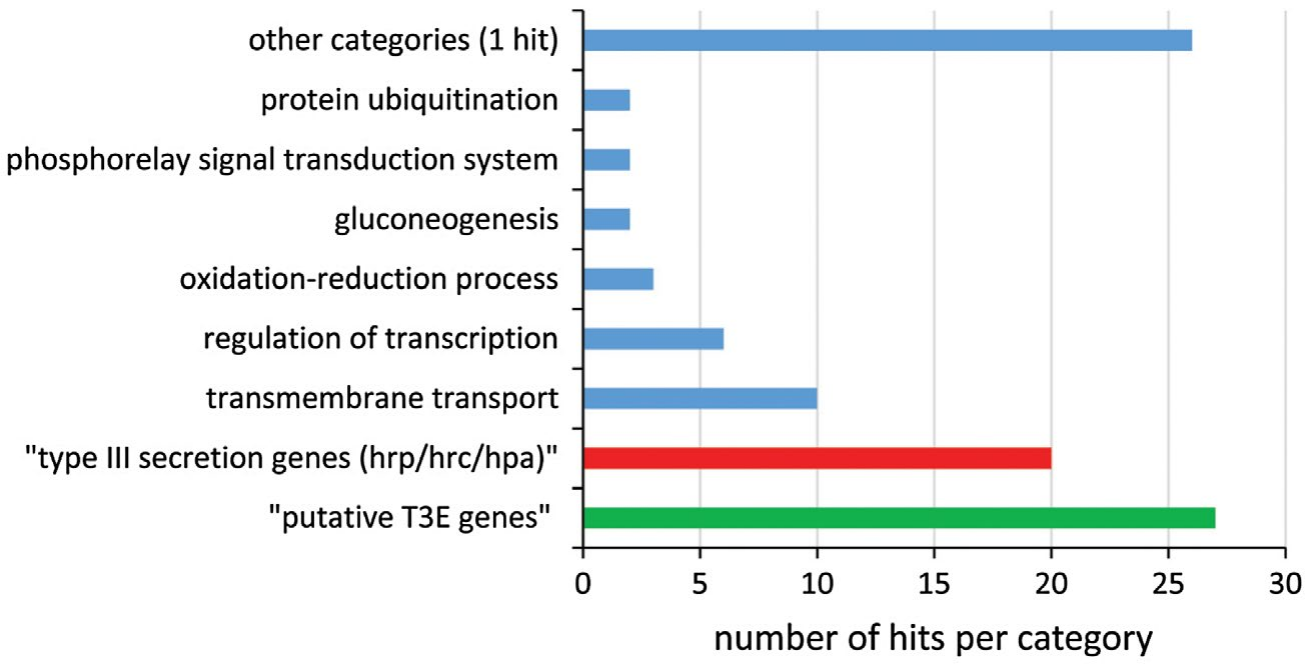
Distribution of *Acidovorax citrulli* M6 HrpX‐regulated genes among categories of biological processes. Of the 159 genes that showed reduced expression in the *hrpX* mutant relative to wild‐type M6, only 47 could be assigned to at least one GO biological process category (blue columns). HrpX‐regulated genes encoding T3S structural and accessory proteins (red column) and putative T3Es (green column) were manually assigned to these categories.

Most HrpX‐regulated genes could not be assigned to gene ontology (GO) categories using Blast2GO. Of the 159 genes with reduced expression in the mutant, only 47 were assigned to at least one biological process category. Blast2GO results are detailed in Table S3C,D, and Fig. 3 shows the number of assigned biological process categories of genes with reduced expression in the mutant. Among the most frequent categories, ten hits were found for transmembrane transport proteins, including several ABC transporters and permeases, and six matched with regulation of transcription. Nine hits belonged to protein secretion/protein secretion by the T3SS (Hrp/Hrc components). Notably, most T3S and T3E genes could not be assigned to any specific GO biological process. This was the case for 11 *hrp*/*hrc*/*hpa* genes and for 24 T3E genes (Table S3C). Overall, RNA‐Seq revealed 20 *hrp*/*hrc*/*hpa* genes and 27 genes encoding putative T3Es (including the seven new effectors identified in this study; see below) that had significantly reduced expression in the *hrpX* mutant relative to wild‐type M6 (Table S3B,C).

The *hrpX* mutant also showed reduced expression of several genes encoding proteins that are putatively secreted by the type II secretion system (T2SS). We used SignalP, Pred‐Tat and Phobius tools to detect Tat or Sec type II secretion (T2S) signals in the ORFs of all genes that showed significantly lower expression in the *hrpX* mutant. While T2S signals were predicted in 39 genes by at least one of the tools (not shown), 14 genes encoded products with T2S signals by the three tools (Table S3E). Among these genes were *APS58_0633* (*xynB*), encoding 1‐4‐β‐xylanase, *APS58_2599* (*pelA_2*), encoding pectate lyase, and *APS58_3722*, encoding a family S1 extracellular serine protease. These three genes also contain PIP boxes in their promoter region (Table S3B).

Of the 28 genes showing increased expression in the *hrpX* mutant relative to wild‐type M6, only ten could be assigned to GO categories, most of which belonged to regulatory genes (Table S3D).

### Identification of PIP boxes in HrpX‐regulated genes

**Figure 4:**
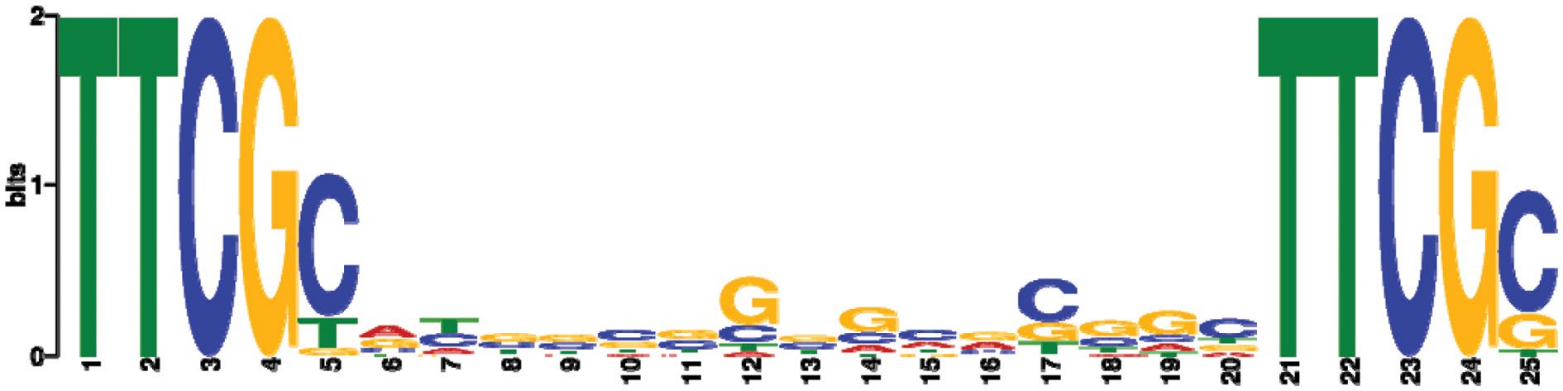
Sequence logo of the *Acidovorax citrulli* M6 PIP box motif. The logo was generated with MEME‐ChiP based on multiple alignment of the 41 perfect PIP boxes that were found to be associated with HrpX‐regulated genes by RNA‐Seq (Table 2).

We used fuzznuc to search for perfect PIP boxes in the *A. citrulli* M6 genome, using the consensus sequence TTCGB‐N15‐TTCGB. This screen revealed a total of 78 PIP boxes (Table S4), of which 41 correlated with significant regulation by HrpX (Tables 2 and S4). We used the PIP boxes of these 41 genes/operons to determine the consensus PIP box of *A. citrulli* (Fig. 4). Importantly, some of the PIP boxes were upstream of operons, thus probably regulating the expression of more than one gene. We detected 25 additional genes [marked as (+) in the PIP box column of Table S3B] that are likely in PIP box‐containing operons and had higher expression in wild‐type M6 relative to the *hrpX* mutant. It is also worth mentioning that 11 additional genes (some of which encode T3Es) carrying PIP boxes showed higher expression values in the wild‐type relative to the *hrpX* mutant in the RNA‐Seq experiment, but were slightly below the threshold of statistical significance (Tables S3A and S4).

**Table 2:** Perfect PIP box sequences in genes that were shown to be regulated by HrpX in *Acidovorax citrulli* M6.

Gene_ID*	Annotation*	Strand	PIP box†	Start of PIP box	End of PIP box	Gene start codon	Distance (bp)‡
*APS58_0030*	*(T3E HopBN1)*	−	ttcgttttgttgattggaaattcgc	34554	34578	34553	1
*APS58_0077*	*HP*	−	ttcgcaattcgagaaatttgttcgg	93187	93211	93022	165
*APS58_0185*	*HP*	−	ttcgtgttgaaggcattcgtttcgg	216423	216447	216315	108
*APS58_0197*	*puuD_1*	−	ttcgtgcatcggctcttccattcgc	227069	227093	226515	554
*APS58_0218*	*HP*	−	ttcgcgtgtgcgtgaactctttcgc	254146	254170	254075	71
*APS58_0500*	*HP*	−	ttcgccccggcctgccggacttcgc	579574	579598	579502	72
*APS58_0502*	*yopJ*	−	ttcgcccggcaggcacccgtttcgc	583025	583049	582809	216
*APS58_0543*	*HP*	−	ttcgcatgcatgtgagcggattcgg	631454	631478	630839	615
*APS58_0633*	*xynB*	−	ttcgcttgctgcttcacgggttcgc	715951	715975	715863	88
*APS58_0886*	*HP*	−	ttcgcatcgccgtgcatggtttcgc	1015878	1015902	1015701	177
*APS58_0986*	*HP*	+	ttcgcattccgcgcgactgcttcgc	1113649	1113673	1113709	36
APS58_1000* §	*HP*	+	ttcgccaccgggcgcacggcttcgt	1129783	1129807	1129817	10
*APS58_1026*	*HP*	−	ttcgtgcacgcgcctgccggttcgc	1161953	1161977	1161806	147
*APS58_1255*	*HP*	+	ttcgcgcgccacggccccgcttcgc	1409795	1409819	1410119	300
*APS58_1340*	*HP*	+	ttcgcatgtccgcggagtcgttcgg	1511860	1511884	1512052	168
*APS58_1448*	*HP*	−	ttcgcgaggccacgcattgcttcgc	1632395	1632419	1632309	86
*APS58_1483*	*HP*	−	ttcgcattcccgtggccggcttcgg	1669735	1669759	1669644	91
*APS58_1760*	*T3E protein*	+	ttcgtgcctgcgggcacgtattcgc	1970321	1970345	1970408	63
*APS58_1954*	*HP*	+	ttcgcaagttctccagctttttcgg	2174442	2174466	2174654	188
*APS58_1986*	*HP*	−	ttcgcgccagcgcgcgggacttcgc	2212320	2212344	2212063	257
*APS58_2304*	*hrcQ*	−	ttcgccttacgcgatgagccttcgg	2546196	2546220	2546073	123
*APS58_2307*	*hrcU*	−	ttcgcgcggggcggaaccgcttcgc	2550224	2550248	2550146	78
*APS58_2308*	*hrpB7*	+	ttcgcattccggtgcgcggcttcgg	2550284	2550308	2550387	79
*APS58_2312*	*hrpW*	+	ttcgcatccgctgcgccgccttcgc	2553056	2553080	2553423	343
*APS58_2314*	*HP*	+	ttcgcgatgccgcatgcagcttcgc	2556756	2556780	2556924	144
*APS58_2329*	*HP*	−	ttcgcaagccatgaagcaacttcgt	2567741	2567765	2566734	7
*APS58_2331*	*hrcC*	−	ttcgcaagccgtcgcgcggcttcgc	2569879	2569903	2569803	76
*APS58_2345*	*(T3E XopP)*	+	ttcgcgcaaaggtgagcggcttcgc	2581209	2581233	2581585	352
*APS58_2347*	*HP*	+	ttcgcaccgccgtgcaggggttcgc	2585239	2585263	2585399	136
*APS58_2599*	*pelA_2*	−	ttcggctgcatggccgccgcttcgc	2862541	2862565	2862501	40
*APS58_2771*	*HP*	+	ttcggaccgctgcgccggcattcgc	3052001	3052025	3052432	407
*APS58_2974*	*HP*	−	ttcgttccaggcaggctgtcttcgc	3262651	3262675	3262590	61
*APS58_3261*	*(T3E HopBF1)*	+	ttcgcctggcgcaatgcgggttcgc	3574633	3574657	3574825	168
*APS58_3289*	*hopW1‐1*	−	ttcgccggggagggcagtttttcgc	3605458	3605482	3605197	261
*APS58_3297*	*HP*	−	ttcgcggggtggcactccgcttcgg	3611673	3611697	3611575	98
*APS58_3344*	*(T3E XopAI)*	−	ttcgcagccctccccggcacttcgc	3656278	3656302	3656147	131
*APS58_3685*	*HP*	−	ttcgcacgttggacatgcatttcgc	3989762	3989786	3989700	62
*APS58_3722*	*HP*	+	ttcgttttaagacgaagaaattcgc	4030992	4031016	4031209	193
*APS58_4095*	*HP*	+	ttcgcatccatggggccggcttcgc	4442672	4442696	4443213	517
*APS58_4116*	*HP*	−	ttcgcgcaggcgcatgcgcgttcgc	4476037	4476061	4475953	84
*APS58_4317*	*(T3E HopD1)*	−	ttcgcaccgtcggccatcgcttcgc	4692889	4692913	4692529	360

* Locus_tag and annotation according to GenBank accession CP029373.1. *HP*, hypothetical protein. Genes showing descriptions between parentheses in the annotation column were annotated as HP but sequence analysis revealed similarity to known T3Es (see Table 1).

† PIP box consensus: TTCGB‐N15‐TTCGB (where B is any nucleotide except adenine).

‡ Distance between the end of the PIP box and the first nucleotide of the start codon.

§ Gene APS58_1000*: this gene was not annotated in the new M6 annotation. It is located between genes *APS58_0999* and *APS58_1000* (positions 1129817–1130383), and its expression was confirmed by RNA‐Seq.

### Evaluation of a translocation assay for validation of *A. citrulli* T3Es

A critical prerequisite for the discovery of new T3Es is the availability of a suitable translocation assay. We assessed the possibility of exploiting the *avrBs2*–*Bs2* gene‐for‐gene interaction to test translocation of predicted *Acidovorax* T3Es into plant cells. The *X. euvesicatoria* AvrBs2 effector elicits an HR in pepper plants carrying the *Bs2* resistance gene (Tai *et al.*, 1999). A truncated form of this effector, carrying amino acids 62–574 (AvrBs2_62‐574_), lacks the N‐terminal translocation signal, but retains the ability to elicit the HR when expressed in *Bs2* pepper cells (Roden *et al.*, 2004a). The *avrBs2–Bs2* translocation assay is thus based on generation of plasmids carrying the candidate T3E (CT3E) genes fused upstream and in frame to AvrBs2_62‐574_. The plasmid is then mobilized into a *X. euvesicatoria* 85‐10 hrpG*Δ*avrBs2* strain that constitutively expresses *hrpG* and lacks *avrBs2*. This strain is used to inoculate leaves of the pepper line ECW20R that carries the *Bs2* gene. If the AvrBs2_62‐574_ domain is fused with a T3E gene, this elicits a *Bs2*‐dependent HR (Roden *et al.*, 2004a; Teper *et al.*, 2016).

**Figure 5:**
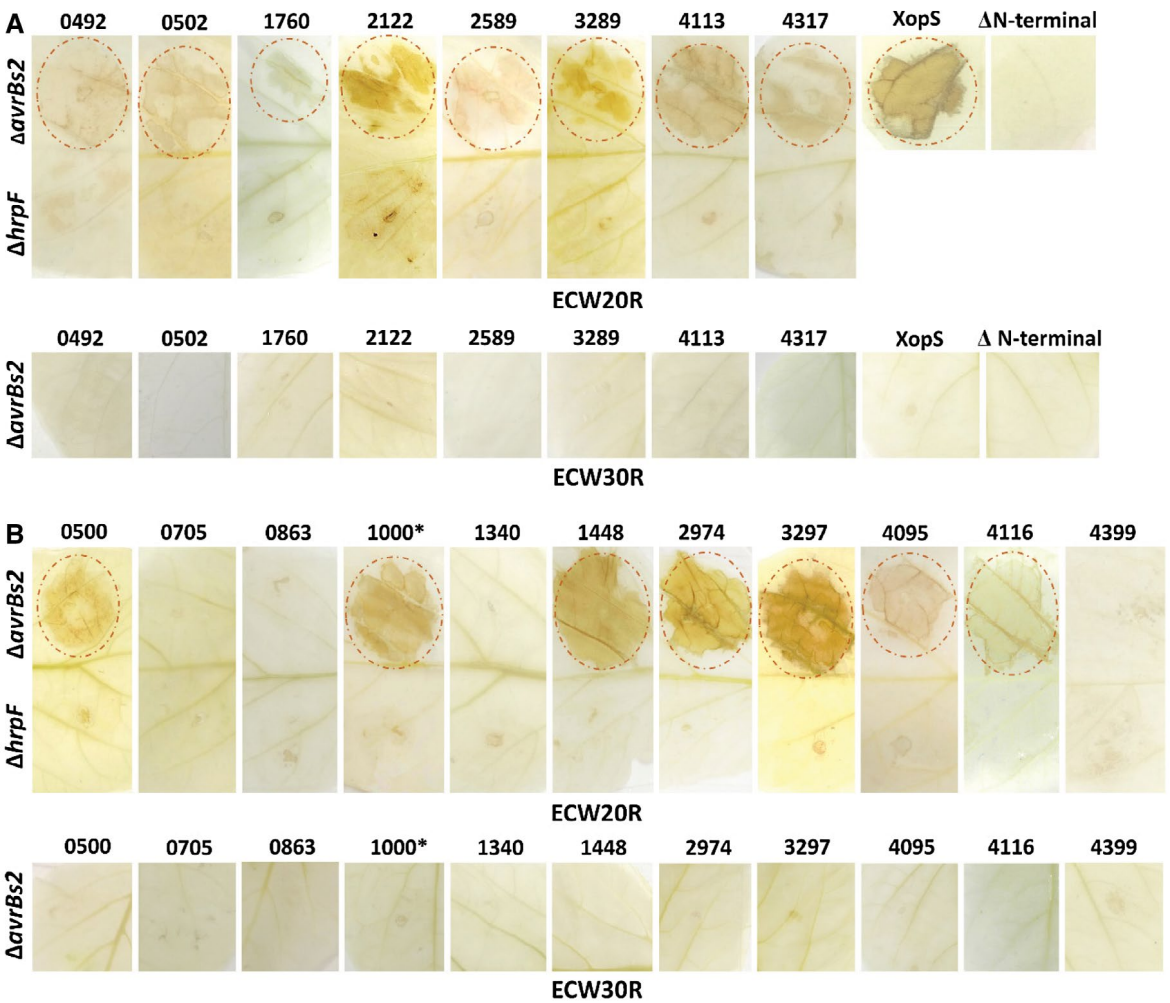
Translocation assays of T3Es of *Acidovorax citrulli* M6. (A) Selected T3Es based on sequence similarity to T3Es from other pathogenic bacteria. (B) Candidate T3Es (CT3Es) selected from machine‐learning and RNA‐Seq. T3E/CT3E ORFs were cloned in plasmid pBBR1MCS‐2 upstream to the AvrBs2_62‐574_ domain, which elicits HR in ECW20R pepper plants carrying the *Bs2* gene, but not in ECW30R pepper plants that lack this gene. The plasmids were transformed into *Xanthomonas euvesicatoria* 85‐10‐hrpG*‐Δ*avrBs2*, and the resulting strains were used to inoculate pepper plants. All known T3Es (A) and seven among 11 tested CT3Es (B) elicited HR in ECW20R leaves but not in ECW30R leaves, similarly to the positive control XopS‐AvrBs2_62‐574_. No HR was induced when leaves were inoculated with a *X. euvesicatoria* mutant impaired in T3S (Δ*hrpF*) expressing T3E/CT3E‐AvrBs2_62‐574_ fusions. Also, no HR was induced following inoculation with *X. euvesicatoria* 85‐10‐hrpG*‐Δ*avrBs2* without any plasmid (not shown) or with a plasmid expressing the AvrBs2_62‐574_ domain alone (ΔN‐terminal). Numbers at the top correspond to the locus_tag in strain M6 (e.g. 0492 is gene *APS58_0492*). Gene APS58_1000* was not annotated in the new annotation of the M6 genome (GenBank accession CP029373.1) but its expression was confirmed by RNA‐Seq (see details in footnote 4 of Table 2).

Given the close similarity between the T3SSs of *A. citrulli* and *Xanthomonas* spp., we hypothesized that the *X. euvesicatoria* T3S apparatus would recognize and translocate *A. citrulli* T3Es, and therefore that the *avrBs2–Bs2* reporter system would be suitable for validating *A. citrulli* CT3E genes. To test this hypothesis, we assessed translocation of the products of eight *A. citrulli* genes with similarity to known T3Es of other pathogenic bacteria. All tested fusions were translocated into pepper cells in a T3S‐dependent manner and induced a *Bs*2‐dependent HR in ECW20R pepper leaves. In contrast, HR was not detected when the fusions were tested in ECW30 leaves (lacking the *Bs2* gene), and when a *X. euvesicatoria hrpF* mutant (impaired in T3S) was used as control (Fig. 5A). With that said, a weak necrosis was detected in ECW20R leaves infiltrated with the *hrpF* mutant carrying fusions of the AvrBs2_62‐574_ domain with effector APS58_2122 (Fig. 5A). While we cannot exclude the possibility that this result was caused by generation of reactive oxygen species due to injury caused by the infiltration treatment (Huang *et al.*, 2019; León *et al.*, 2001), it is possible that this effector possesses additional, T3S‐independent export signals that allow its entering into the plant cell, as recently shown for the *X. euvesicatoria* effector AvrBs3 (Scheibner *et al.*, 2017).

### Validation of seven novel T3Es of *A. citrulli*

Following validation of the *avrBs2–Bs2* reporter assay for *A. citrulli* T3Es, we selected seven CT3Es based on ML and RNA‐Seq results. Four genes that were ranked relatively low in the ML were also included in these experiments (Tables 3 and S1). All seven CT3E genes, but not the low‐ranked ML genes, were translocated (Fig. 5B). The validated genes were annotated as hypothetical proteins, had predicted PIP boxes, were shown to be positively regulated by HrpX and ranked high in the ML run (Tables 3 and S1). Importantly, the gene *APS58_1340*, which contains a PIP box in its promoter region and has higher expression in wild‐type M6 than in the *hrpX* mutant (Table 3), was not translocated, indicating that these two parameters alone are not sufficient for accurate prediction of T3Es*.*

**Table 3:** Candidate T3E genes of *Acidovorax citrulli* M6 that were tested in the *avrBs2*–*Bs2* translocation assays.

Gene ID*	Product	ML†	PIP‡	RSEQ§	TRA¶
**Genes that were ranked relatively high in ML1**					
***APS58_0500***	Hypothetical protein**	39/48	+	+	+
APS58_1000*	Hypothetical protein**	21/*	+	+	+
***APS58_1448***	Hypothetical protein**	17/104	+	+	+
***APS58_2974***	Hypothetical protein**	19/29	+	+	+
***APS58_3297***	Hypothetical protein**	20/61	+	+	+
***APS58_4095***	Hypothetical protein**	31/46	+	+	+
***APS58_4116***	Hypothetical protein**	11/43	+	+	+
**Genes that were ranked relatively low in ML1**					
***APS58_0705***	GrxD, glutaredoxin‐4	91/2203	−	−	−
***APS58_0863***	Hypothetical protein	64/749	−	−	−
***APS58_1340***	Hypothetical protein	84/535	+	+	−
***APS58_4399***	Hypothetical protein	174/739	−	−	−

* Gene IDs are according to the annotation of the *A. citrulli* M6 chromosome (GenBank accession CP029373.1).

† ML: rankings in first/second machine‐learning runs. *, gene APS58_1000* was not included in the second ML run as it was not annotated (see footnote 4 of Table 2).

‡ PIP: presence (+) or absence (−) of perfect PIP box in the promoter region.

§ RSEQ: significantly reduced expression in the *hrpX* mutant relative to the wild‐type (+) or no significant differences between strains (−).

¶ TRA: translocated (+) or non‐translocated (−) in *avrBs2–Bs2* translocation assays.

** These genes were detected only in plant pathogenic *Acidovorax* species.

Interestingly, BLAST analyses of the seven new T3E genes revealed strong similarity only to hypothetical proteins of plant pathogenic *Acidovorax* species. The fact that no homologues for these genes were detected in nonpathogenic *Acidovorax* strains (despite the availability of more than 70 genomes of such species) or in other plant pathogenic bacterial species suggests a specific and unique role for their products in *Acidovorax* pathogenicity. These seven genes were detected also in AAC00‐1 (Table S2) and in all other group I and II genomes available in NCBI. Some of them were widely distributed among other plant pathogenic *Acidovorax* species (Table S2). Searches for conserved domains in these T3Es did not provide any functional insight.

### Assessment of localization of three of the new T3Es

Prediction of subcellular localization of effectors APS58_0500, APS58_1448 and APS58_4116 using the Plant‐mPLoc server indicated that they could localize to the plant cell nucleus. Browsing these T3Es with the LogSigDB server revealed endoplasmic reticulum (ER) localization signals in the three effectors, and nuclear localization signals in APS58_0500 and APS58_4116.

**Figure 6:**
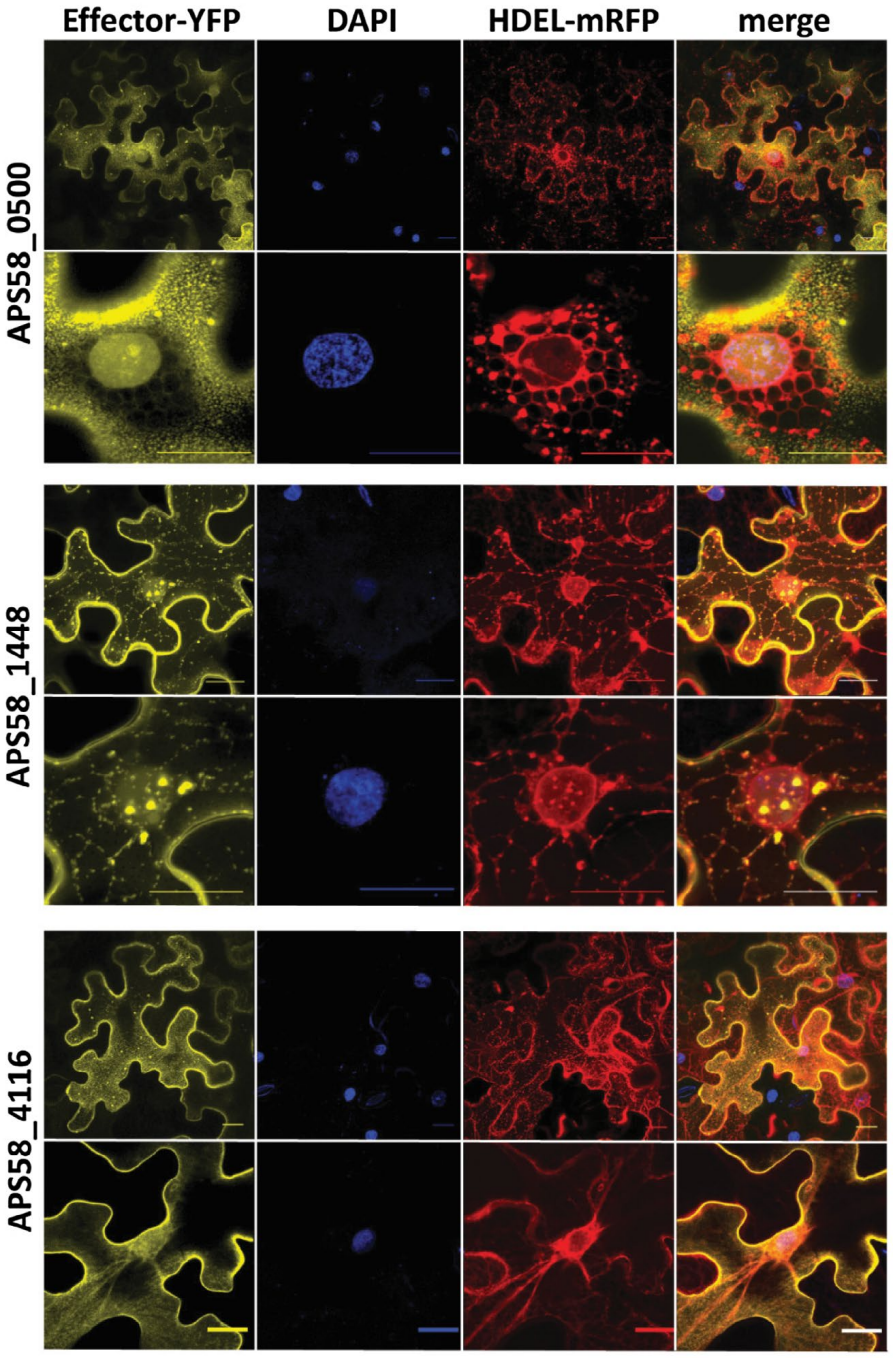
Transient expression of *Acidovorax citrulli* T3Es in *Nicotiana benthamiana*. T3E genes *APS58_0500*, *APS58_4116* and *APS58_1448* were cloned in pEarleyGate101, fused to the C‐terminus of YFP. The plasmids were transformed into *Agrobacterium tumefaciens* GV3101, and the resulting strains were used for transient expression in *N. benthamiana*. Leaves were co‐inoculated with *A. tumefaciens* GV3101 carrying the mRFP‐HDEL endoplasmic reticulum marker and stained with DAPI for visualization of plant cell nuclei. Samples were visualized in a Leica SPE confocal microscope 48 h after inoculation. For each T3E, two sets of images are shown, with the lower images being magnifications of selected areas of the upper images. Bars at the right bottom of each picture, 20 µm.

We assessed localization of these effectors fused to the yellow fluorescent protein (YFP) in *Nicotiana benthamiana* following transient expression by agroinfiltration. Based on the aforementioned predictions, in the first experiments the leaves were co‐infiltrated with *Agrobacterium tumefaciens* carrying the ER marker mRFP‐HDEL and were also stained with 4′,6‐diamidine‐2′‐phenylindole dihydrochloride (DAPI) for nucleus localization. Representative images from these experiments are shown in Fig. 6. The results indicate that the three effectors could interact with the ER, but only APS58_0500 and APS58_1448 partially localized to the nucleus, including in clearly visible nuclear foci (Fig. 6).

In a second set of experiments, the YFP‐fused effectors were co‐infiltrated with free‐mCherry, localized mainly in the cytosol and in the nucleus, HDEL‐mCherry, localized to the ER, and the membrane‐bound protein SlDRP2A (L. Pizarro and M. Bar, unpublished results). Representative images from these experiments are shown in Figs S2 to S4 for APS58_0500, APS58_1448 and APS58_4116, respectively. The three effectors partially co‐localized with the membrane‐bound protein SIDRPA, as evidenced by the Pearson correlation coefficients (0.40 ± 0.024 for APS5_0500, 0.49 ± 0.040 for APS58_1448 and 0.53 ± 0.037 for APS58_4116). Since APS58_0500 appeared to have a stronger membrane localization, we used the classical plasma membrane microdomain protein Flot1 (Li *et al.*, 2012) as an additional membrane control marker. Indeed, APS58_0500 had an expression pattern that was highly similar to that of Flot1 (Fig. S2). In agreement with the first set of experiments (Fig. 6), APS58_0500 (Fig. S2) and APS58_1448 (Fig. S3) partially localized to the nucleus. On the other hand, these experiments confirmed that only APS58_4116 partially interacted with the ER, mostly at the nuclear envelope (Figs 6 and S4; Pearson coefficient with HDEL‐mCherry 0.53 ± 0.037). None of the effectors has a significant cytosolic presence: the Pearson coefficient with mCherry was lower than 0.12 for APS_0500 and APS_4116, while for APS_1448 the coefficient was 0.53 ± 0.037 due to the strong nuclear presence of this effector, as indicated above. Overall, we conclude that the three effectors are associated with the plasma membrane, APS58_0500 and APS58_1448 partially localize to the nucleus, and APS_4116 partially interacts with the ER.

### Generating an improved list of *A. citrulli* M6 CT3Es with a second ML run

Since ML can be improved after refinement of features specific to the studied pathogen, we carried out a second ML run. The main differences between the first and second ML runs were (i) the second run was done on the M6 genome, which by this time was fully assembled (Yang *et al.*, 2019), (ii) we added the seven novel T3Es identified in this study and the four ORFs that were found not to be translocated to the positive and negative sets, respectively, (iii) in the positive set we included ORFs with high sequence similarity to known effectors from other bacteria, based on our homology search results (Table 1), and (iv) we used HrpX‐mediated regulation as an additional feature to train the classifier. The results of the second ML run are summarized in Table S5. Most known/validated T3Es ranked among the top 100 hits and among the top 40 hits, 34 were known/validated T3Es. Importantly, some genes with high propensity to encode T3Es (ranking among the top 60 in the second ML run) did not appear among the top 200 hits in the first ML list (Tables 1 and S1), thus supporting the higher reliability of the new list relative to the first prediction.

Among the top 100 hits of the second ML run, there were 37 genes that matched to hypothetical proteins, with no similarity evidence to suggest a T3E nature. Since this was the case of the seven novel T3Es validated in this study, it is possible that some of these genes encode yet undiscovered T3Es. In this regard, it is worth mentioning genes *APS58_1954*, *APS58_1986*, *APS58_3685*, *APS58_0987* and *APS58_1694* (ranking at positions 20, 27, 57, 62 and 83 in the second ML, respectively). While *APS58_1694* shares similarity only with hypothetical proteins of plant pathogenic *Acidovorax* species, the others also share similarities to hypothetical proteins of other plant pathogenic genera (e.g. *Xanthomonas*, *Ralstonia*, *Pseudomonas* and/or *Erwinia*). These genes also had increased expression in wild‐type M6 relative to the *hrpX* mutant and have PIP boxes in their promoter region. These genes are therefore strong candidates for further experimental validations.

## Discussion

*Acidovorax citrulli* requires a functional T3SS for pathogenicity (Bahar and Burdman, 2010). The main objective of this study was to significantly advance the current knowledge about the arsenal of T3Es of *A. citrulli*. Among well‐investigated plant pathogenic bacteria, the pools of T3Es vary from only few effectors in phytopathogenic bacteria from the *Enterobacteriaceae* family (Hogan *et al.*, 2013; Nissinen *et al.*, 2007) to approximately 20–40 in most strains of *P. syringae* and *Xanthomonas* spp. (Büttner and Bonas, 2010; Chang *et al.*, 2005; Kvitko *et al.*, 2009; O'Brien *et al.*, 2011; Schechter *et al.*, 2006; Teper *et al.*, 2016; White *et al.*, 2009) and an average of over 75 in *R. solanacearum* isolates (Deslandes and Genin, 2014; Peeters *et al.*, 2013). Thus, we hypothesized that the repertoire of *A. citrulli* T3Es could be much larger than the 11 T3E genes reported in the group II strain AAC00‐1 (Eckshtain‐Levi *et al.*, 2014).

As a first approach to uncover the *A. citrulli* T3E arsenal, we used a genome‐wide ML algorithm to determine the propensity of ORFs to encode T3Es. In parallel, we looked carefully at the annotation of the group I model strain of *A. citrulli*, M6, and carried out BlastP analyses of genes encoding hypothetical proteins or functions that could infer effector activity. These analyses revealed 51 putative T3E genes that shared different levels of similarity with known effector genes from *Xanthomonas* spp., *R. solanacearum* and/or *P. syringae* strains (Table 1). Homologues for most of these T3E genes and for those identified in the present study were also detected in other plant pathogenic *Acidovorax* species (Table S2).

To identify new putative T3Es of *A. citrulli*, we also used RNA‐Seq to identify HrpX‐regulated genes. Based on the knowledge accumulated with *Xanthomonas* spp. and *R. solanacearum* (Genin and Denny, 2012; Guo *et al.*, 2011; Koebnik *et al.*, 2006; Occhialini *et al.*, 2005), we expected that most genes encoding T3SS components and some T3Es of *A. citrulli* would be under the direct regulation of HrpX. This assumption was strengthened in preliminary experiments comparing gene expression between wild‐type M6 and a *hrpX* mutant (Fig. 1C). As previously mentioned, Zhang *et al. *(2018) recently showed that HrpX controls the expression of one T3E gene in the group II strain, Aac5.

RNA‐Seq revealed 159 genes showing significantly reduced expression in the *hrpX* mutant, while 28 genes had significantly increased expression in the mutant (Table S3). These numbers are similar to those reported in gene expression studies carried out with *Xanthomonas* spp. HrpX and with *R. solanacearum* HrpB. For instance, microarray analyses of *Xanthomonas axonopodis* pv. *citri* (*Xac*) revealed that 181 genes were up‐regulated by HrpX, while 5–55 genes (depending on the time point) were down‐regulated by this transcriptional regulator (Guo *et al.*, 2011). Occhialini *et al. *(2005) found 143 HrpB up‐regulated genes and 50 HrpB down‐regulated genes in *R. solanacearum*. In these, as well as in other studies, HrpX/HrpB was found to regulate the expression of most genes encoding T3S components and accessory proteins as well as several T3E genes (Büttner and Bonas, 2010; Genin and Denny, 2012; Valls *et al.*, 2006). In line with this background, among the 159 HrpX up‐regulated genes found in our study, 20 encoded *hrp*/*hrc*/*hpa* genes and 27 encoded T3E genes. Interestingly, *hrcC* was a member of the *A. citrulli* HrpX regulon. *hrcC* expression in *X. euvesicatoria* is directly regulated by HrpG, in an HrpX‐independent manner (Wengelnik *et al.*, 1996b). In contrast, in *R. solanacearum*, *hrcC* is regulated by HrpX (Brito *et al.*, 1999; Valls *et al.*, 2006), as we found in *A. citrulli* M6.

In *Xanthomonas* spp. and in *R. solanacearum*, the HrpX/HrpB regulons include genes that are not involved in T3S (Büttner and Bonas, 2010; Guo *et al.*, 2011; Valls *et al.*, 2006). A similar picture emerged from our study, where HrpX was shown to regulate genes involved in transmembrane transport, including several ABC transporters and permeases as well as transcriptional regulators. Among the HrpX up‐regulated genes we also detected several genes whose products are putatively secreted by the T2SS. These included genes encoding 1,4‐β‐xylanase, pectate lyase and a protein similar to S1 extracellular serine proteases (Table S3E). HrpX regulation of genes encoding type II‐secreted enzymes was also demonstrated in *Xanthomonas* spp. and in *R. solanacearum* (Furutani *et al.*, 2004; Genin and Denny, 2012; Guo *et al.*, 2011; Szczesny *et al.*, 2010; Wang *et al.*, 2008; Yamazaki *et al.*, 2008).

More than 60 HrpX up‐regulated genes carried perfect PIP boxes in their promoter region or were part of operons carrying perfect PIP boxes (Tables 2, S3B and S4). Although some other genes may carry imperfect PIP boxes and may be directly regulated by HrpX, this result suggests that many of the HrpX up‐regulated genes are indirectly regulated by this transcriptional factor. This is a reasonable assumption, considering that among the genes that were up‐ and down‐regulated by HrpX there were several transcriptional regulators. For instance, genes encoding transcriptional factors belonging to the LysR (*APS58_0949* and *APS58_2039*), IclR (*APS58_1263*), FmbD (*APS58_1340*) and TetR (*APS58_3638*) families were up‐regulated by HrpX. In contrast, two genes encoding DNA‐binding response regulators, homologous to PhoP (*APS58_0821*) and FixJ (*APS58_1682*), were HrpX down‐regulated (Table S2B).

After demonstrating the suitability of the *avrBs2–Bs2* T3E translocation assay, we used the data obtained from ML and RNA‐Seq to select seven *A. citrulli* M6 CT3Es for experimental validation. We validated translocation of the seven candidates (Fig. 5), thus demonstrating the strength of combining these approaches for identifying new T3E genes. Importantly, the lack of translocation of the four ORFs that received relatively low scores in the ML strengthened the suitability of our combined computational/experimental approach.

An interesting trait of the seven new T3Es is that they share significant similarity only with hypothetical proteins of other plant pathogenic *Acidovorax* strains (Tables 3 and S2). These effectors may be involved in the pathoadaptive evolution of plant pathogenic *Acidovorax* species. Importantly, a second ML run, informed by the knowledge accumulated from this study, revealed additional genes that were ranked in relatively high positions and encoded hypothetical proteins that occur only in plant pathogenic *Acidovorax* or in other plant pathogenic bacteria (Table S5). These represent high‐priority CT3Es for future experimental validation assays. This result also emphasizes one benefit of the ML approach: its ability to integrate novel knowledge in the prediction algorithm. Importantly, since some of these new CT3E candidates share similarity with hypothetical proteins of other plant pathogenic bacterial species, this information could be exploited to identify new T3Es in other bacterial pathogens.

Another interesting characteristic of the new T3Es discovered in this study is their relatively small size. Based on the annotation of the M6 genome, the mean and median lengths of *A. citrulli* M6 T3Es are 387.7 and 345 amino acids, respectively. Except for *APS_4116*, which encodes a 347 amino acid protein, the predicted size of the six other new T3Es ranged from 113 amino acids (APS58_4095) to 233 amino acids (APS58_0500) (Fig. S5). In the public database (GenBank), there are several examples of small T3Es from plant pathogenic bacteria, including *Xanthomonas* AvrXv3 (most having 119 amino acids), *P. syringae* HopAF1 (112–291 amino acids), HopBF1 (125–207 amino acids), HopF2 (177–280 amino acids), HopH1 (201–218 amino acids) and AvrRpt2 (222–255 amino acids), and the *R. solanacearum*/*Xanthomonas* HopH1 homologues (155–218 amino acids).

In this study we assessed plant cell localization of three of the new T3Es validated in translocation assays, APS58_0500, APS58_1448 and APS58_4116. Utilization of subcellular localization prediction tools and confocal microscopy of *N. benthamiana* agroinfiltrated leaves strongly suggest that the three tested effectors interact with the plasma membrane (Figs S2–S4), with APS58_0500 remarkably mimicking the localization of the classical non‐clathrin mediated endocytic system protein, Flot1 (Li *et al.*, 2012). While APS58_4116 interacted with the ER (Figs 6 and S2), effectors APS58_0500 and APS58_1448 partially localized to the nucleus (Figs 6, S3 and S4). Further characterization of the novel T3Es identified in this study may uncover new host targets of pathogen effectors and new mechanisms by which pathogenic bacteria manipulate their hosts.

In conclusion, we have combined sequence analysis, ML and RNA‐Seq approaches to uncover the arsenal of T3Es of the group I model strain of *A. citrulli*, M6, including discovery of new T3Es that appear to be unique to plant pathogenic *Acidovorax* spp. We also demonstrated the suitability of a translocation reporter system for validation of *A. citrulli* T3Es, which we expect will be very helpful to the *Acidovorax* research community. Until recently it was assumed that *A. citrulli* strains (and plant pathogenic *Acidovorax* strains in general) possess little over ten T3E genes. However, this study revealed that the *A. citrulli* pan‐genome encodes more than 50–60 T3Es, placing this pathogen among the ‘richest’ bacteria in terms of T3E cargo. Remarkably, a second ML run strongly suggests that *A. citrulli* may possess yet unrevealed T3E genes.

## Experimental Procedures

### Bacterial strains and plasmids

The bacterial strains and plasmids used in this study are listed in Table S6. Unless stated otherwise, *A. citrulli* strains were grown at 28 °C in nutrient broth (NB; Difco Laboratories, Detroit, MI, USA) or nutrient agar (NA; NB containing 15 g/L agar). For RT‐PCR, qRT‐PCR and RNA‐Seq experiments, *A. citrulli* strains were grown in XVM2 medium (Wengelnik *et al.*, 1996b). *Xanthomonas euvesicatoria*, *A. tumefaciens* and *E. coli* strains were cultured on Luria‐Bertani (LB) medium (Sambrook *et al.*, 1989) at 28 °C for *X. euvesicatoria* and *A. tumefaciens*, and 37 °C for *E. coli*. When required, media were supplemented with the following antibiotics: ampicillin (Ap, 100 µg/mL for *E. coli* and 200 µg/mL for the others), rifampicin (Rif, 50 µg/mL), kanamycin (Km, 50 µg/mL) and gentamicin (Gm, 50 µg/mL for *A. citrulli* and 10 µg/mL for the others).

### Molecular manipulations

Routine molecular manipulations and cloning procedures were carried out as described in Sambrook *et al.* (1989). T4 DNA ligase and restriction enzymes were purchased from Fermentas (Burlington, Canada). AccuPrep Plasmid Mini Extraction Kit and AccuPrep PCR Purification Kit were used for plasmid and PCR product extraction and purification, respectively (Bioneer Corporation, Daejeon, Republic of Korea). DNA was extracted with the GeneElute bacterial genomic DNA Kit (Sigma‐Aldrich, St Louis, MO, USA). PCR primers were purchased from Sigma‐Aldrich and are listed in Table S7. PCRs were performed with the Readymix Red Taq PCR reactive mix (Sigma‐Aldrich) or with the Phusion high‐fidelity DNA polymerase (Fermentas, Waltham, MA, USA) using an Eppendorf (Hamburg, Germany) thermal cycler. Sequencing of PCR fragments and constructs was performed at Hy Laboratories (Rehovot, Israel). *Escherichia coli* strains were transformed using an Eppendorf 2510 electroporator according to manufacturer's instructions. Plasmid mobilizations to *A. citrulli* and *X. euvesicatoria* strains were done by biparental mating as described (Bahar *et al.*, 2009). *Agrobacterium tumefaciens* cells were transformed by the heat shock method (Zhou *et al.*, 2009).

### Machine‐learning classifications

In order to predict T3Es, we applied ML classification algorithms, which are similar to the ones we have previously described (Burstein *et al*., 2009; Lifshitz *et al*., 2014; Nissan *et al.*, 2018; Teper *et al*., 2016). The first ML run was used to search for T3Es in the AAC00‐1 genome (GenBank accession CP000512.1). The training data included 12 ORFs that were known as T3Es (see the Results section). The negative set included 2680 ORFs that had high similarity (*E* < 0.001) to ORFs in the nonpathogenic *E. coli* K12 genome (accession number NC_000913.3). The positive and negative ORFs are marked in Table S1. For this ML, 71 features were used, including homology (to known effectors or to bacteria without T3SS), composition [amino acid composition, guanine + cytosine (GC) content], location in the genome (e.g. distance from known T3Es) and the presence of a PIP box in the promoter region. The complete list of features is given in Table S8. Features were extracted using in‐house Python scripts. The outcome of the ML run is a score for each ORF, reflecting its likelihood to encode a T3E. We evaluated several classification algorithms: random forest (Breiman, 2001), naïve Bayes (Langley *et al*., 1992), support vector machine (SVM; Burges, 1998), K nearest neighbours (KNN), linear discriminate analysis (LDA), logistic regression (all three described in Hastie *et al.*, 2001), and Voting, which aims to predict averaging over all other ML algorithms. For each run, feature selection was performed. The ML algorithms and feature selection were based on the Scikit‐learn module in Python (Pedregosa *et al.*, 2011). The area under the curve (AUC) score over 10‐fold cross‐validation was used as a measure of the classifier performance. The first ML run was based on the random forest classifier, which gave the highest AUC (0.965). The second ML run was similar to the first, but it was run on the M6 genome (GenBank accession CP029373.1) and included additional information as described in the Results section. This run was based on Voting classifier, which included all the classifiers specified above as it gave the highest AUC among all the classifiers. The AUC for this second ML run was 0.999.

### Generation of *A. citrulli* mutants and complemented strains

*Acidovorax citrulli* M6 mutants disrupted in *hrpX* (*APS58_2298*) and *hrpG* (*APS58_2299*) genes were generated by single insertional mutagenesis following single homologous recombination. Internal fragments of the *hrpX* (383 bp) and *hrpG* (438 bp) ORFs carrying nucleotide substitutions that encode early stop codons were PCR‐amplified and inserted into the *Bam*HI/*Eco*RI site of the suicide plasmid pJP5603 (Penfold and Pemberton, 1992). The resulting constructs were transformed into *E. coli* S17‐1 λpir, verified by sequencing, and mobilized into *A. citrulli* M6 by biparental mating. Transconjugants were selected by Km selection. Disruption of the target genes by single homologous recombination and plasmid insertion was confirmed by PCR and sequencing of amplified fragments. To generate complemented strains for mutants disrupted in *hrpX* and *hrpG* genes, the full ORFs of these genes (1407 and 801 bp, respectively) were PCR‐amplified and cloned into the *Eco*RI/*Bam*HI sites of pBBR1MCS‐5 (Kovach *et al.*, 1995). The generated plasmids were transformed into *E. coli* S17‐1 λpir, verified by sequencing and transferred by biparental mating into the corresponding M6 mutant strains. Complemented strains were selected by Gm resistance and validated by PCR.

### Infiltration of melon and pepper leaves with *A. citrulli* strains

Melon (*Cucumis melo*) cv. HA61428 (Hazera Genetics, Berurim, Israel) plants were grown in a greenhouse at *c*. 28 °C. Pepper (*Capsicum annum*) cv. ECW20R and ECW30 (Kearney and Staskawicz, 1990) plants were grown in a growth chamber (16 h/26 °C in the light, 8 h/18 °C in the dark, relative humidity set to 70%). The three youngest, fully expanded leaves of 3‐week‐old melon and 5‐week‐old pepper plants were syringe‐infiltrated in the abaxial side with bacterial suspensions of *A. citrulli* strains containing 10^8^ colony‐forming units (cfu)/mL in 10 mM MgCl_2_. Phenotypes were recorded 3 and 4 days after inoculation (dai) for melon and pepper leaves, respectively. For a better visualization of HR symptoms in pepper leaves, the infiltrated leaves were bleached by soaking them in an acetic acid:glycerol:water solution (1:1:1 v/v/v) for 4 h. The leaves were then transferred to ethanol and boiled for 10 min. Experiments were repeated twice with similar results.

### RNA isolation, cDNA synthesis and RT‐PCR

*Acidovorax citrulli* M6 and *hrpX* mutant were grown at 28 °C in 5 mL of XVM2 medium for 72 h with shaking (180 rpm). Total RNA was isolated using TRI reagent (Sigma‐Aldrich) and Direct‐zol RNA miniprep kit (Zymo Research, Irvine, CA, USA) according to manufacturer's instructions. Samples were treated with RNase‐free DNase using Turbo DNA‐free kit (Invitrogen, Carlsbad, CA, USA). RNA concentration was quantified using a NanoDrop DS‐11 FX (Denovix, Wilmington, DE, USA) and RNA integrity was assayed on 1% agarose gels. RNA was reverse transcribed into cDNA using a High Capacity cDNA Reverse Transcription Kit (Applied Biosystems, Waltham, MA, USA). Semiquantitative RT‐PCR analysis was performed using 1 μg of cDNA or gDNA (as positive control for amplification), 0.6 pmol of selected primer, the Phusion High‐Fidelity DNA Polymerase (ThermoFisher Scientific, Waltham, MA, USA), and the following conditions: 98 °C for 15 min, followed by 35 cycles of 98 °C for 30 s, 60 °C for 30 s and 72 °C for 15 s. The *A. citrulli GAPDH* housekeeping gene (Shavit *et al.*, 2016) was used as reference. The relative amount of amplified DNA was assayed on 2% agarose gels.

### RNA‐Seq and quality analysis

Total RNA of wild‐type M6 and *hrpX* mutant strains was isolated as described above for RT‐PCR experiments. Three independent RNA extractions were obtained for each strain. Ribosomal RNA was depleted using the MICROB Express Bacterial mRNA Purification Kit (Ambion, Foster City, CA, USA). The integrity and quality of the ribosomal depleted RNA was checked by an Agilent 2100 Bioanalyzer chip‐based capillary electrophoresis machine (Agilent Technologies, Santa Clara, CA, USA). RNA sequencing was carried out at the Center for Genomic Technologies at The Hebrew University of Jerusalem (Jerusalem, Israel). The samples were used to generate whole transcriptome libraries using the NextSeq 500 high output kit (Illumina, San Diego, CA, USA) with a NextSeq 2000 sequencing instrument (Illumina). The cDNA libraries were quantified with a Qubit 2.0 fluorometer (Invitrogen) and their quality was assessed with an Agilent 2200 TapeStation system (Agilent Technologies). One of the *hrpX* mutant libraries was removed from further analysis due to low quality. Raw reads (fastq files) were further inspected with FastQC v. 0.11.4 (Martin, 2011). They were trimmed for quality and adaptor removal using Trim Galore default settings: trimming mode single‐end; Trim Galore v. 0.4.3, Cutadapt v. 1.12, Quality Phred score cut‐off 20, quality encoding type selected ASCII + 33, adapter sequence AGATCGGAAGAGC (Illumina TruSeq, Sanger iPCR; auto‐detected), maximum trimming error rate 0.1, minimum required adapter overlap (stringency) 1 bp. An average of 0.6% of the reads were quality trimmed and 57% of the reads were treated for adaptor removal.

### Mapping of RNA‐Seq reads on the *A. citrulli* M6 genome and differential expression analysis

Cleaned reads (*c*. 20 million per sample) were mapped against the latest version of the *A. citrulli* M6 genome (CP029373.1) using STAR v. 2.201 (Dobin *et al.*, 2013). Mapping files were further processed for visualization by Samtools Utilities v. 0.1.19 (Li *et al.*, 2009). The resulting Bam files were used to improve gene and operon predictions along the genome using cufflinks v. 2.2.1 followed by cuffmerge without a guiding reference file (Trapnell *et al.*, 2010). Uniquely mapped reads per gene were counted twice [once using the original submitted annotation file (orig.gff) and then using the merged annotations by cufflinks‐cuffmerge (merged.gff)] using HTSeq‐count (Ander and Hubert, 2010). Differential expression analysis was performed using the DESeq2 R package (Ander and Hubert, 2010). Differentially expressed genes were defined as those genes with a fold‐change higher than 2 and a *P*‐value lower than 0.05.

### Validation of RNA‐Seq results by quantitative real‐time PCR

RNA‐Seq data were verified by qRT‐PCR using specific primers of selected genes (Table S7). Bacterial growth, RNA isolation and cDNA synthesis were as described above for RT‐PCR and RNA‐Seq experiments. qRT‐PCRs were performed in a Light Cycler 480 II (Roche, Basel, Switzerland) using 1 μg cDNA, 0.6 pmol of each primer and the HOT FIREPol EvaGreen qPCR Mix Plus (Solis BioDyne, Tartu, Estonia), and the following conditions: 95 °C for 15 min (1 cycle); 95 °C for 15 s, 60 °C for 20 s and 72 °C for 20 s (40 cycles); melting curve profile from 65 to 97 °C to verify the specificity of the reaction. The *A. citrulli GAPDH* gene was used as an internal control to normalize gene expression. The threshold cycles (*C*_t_) were determined with the Light Cycler 480 II software (Roche) and the fold‐changes of three biological samples with three technical replicates per treatment were obtained by the ΔΔ*C*_t_ method (Pfaffl, 2001). Significant differences in expression values were evaluated using the Mann–Whitney nonparametric test (α = 5%).

### Additional bioinformatics tools

BlastP analyses to search T3E homologues were done at the NCBI server against the non‐redundant protein sequences (nr) database, selecting the organisms *Acidovorax* (taxid: 12916), *Xanthomonas* (taxid: 338), *Ralstonia* (taxid: 48736) or *P. syringae* group (taxid: 136849), with default parameters. GO assignments were done using Blast2GO software v. 5.2 (https://www.blast2go.com/). SignalP v. 4.1 (Petersen *et al.*, 2011), Phobious (Käll *et al.*, 2007) and Pred‐Tat (Bagos *et al.*, 2010) were used for detection of N‐terminal type II secretion signal peptides. The program fuzznuc (EMBOSS package; http://www.bioinformatics.nl/cgi-bin/emboss/fuzznuc) was used to detect perfect PIP box sequences (TTCGB‐N15‐TTCGB; Koebnik *et al.*, 2006) in the *A. citrulli* M6 genome. A logo of the PIP box motif of *A. citrulli* M6 was made with MEME‐ChiP (Machanick and Bailey, 2011) at the MEME Suite website (http://meme-suite.org/). Domain search of T3Es was carried out using the following databases/tools: Protein Data Bank (PDB) and UniProtKB/Swiss‐Prot (through NCBI Blast), PFAM (https://pfam.xfam.org/), Prosite (https://prosite.expasy.org/) and InterPro (https://www.ebi.ac.uk/interpro/search/sequence-search). LogSidDB (Negi *et al.*, 2015) and Plant‐mPLoc (Chou and Shen, 2008) were used for detection of protein localization signals and for prediction of subcellular localization of T3Es, respectively.

### Translocation assays

The ORFs without the stop codon of candidate genes were amplified using specific primers (Table S7) and cloned into the *Sal*I/*Xba*I sites of pBBR1MCS‐2*::avrBs2*_62‐574_, upstream to and in frame with the *avrBs2*_62‐574_ HR domain of *avrBs2* and a haemagglutinin (HA) tag (Teper *et al.*, 2016), except for ORFs of genes *APS58_0500* and *APS58_1760*, which were cloned into the *Xho*I/*Xba*I sites of the same vector. The resulting plasmids were mobilized into *X. euvesicatoria* strains 85‐10 hrpG*Δ*avrBs2* (Roden *et al.*, 2004a) and 85‐10 hrpG*Δ*hrpF* (Casper‐Lindley *et al.*, 2002). Expression of recombinant T3E/CT3E‐AvrBs2_62‐574_‐HA proteins was verified by western blot using the iBlot Gel Transfer Stacks Nitrocellulose kit (Invitrogen), and anti‐haemagglutinin (HA)‐tag and horseradish peroxidase (HRP) antibodies (Cell Signaling Technology, Danvers, MA, USA) (Fig. S6). For translocation assays, *X. euvesicatoria* strains were grown overnight in LB broth with Km, centrifuged and resuspended in 10 mM MgCl_2_ to a concentration of 10^8^ cfu/mL. These suspensions were used to infiltrate the three youngest, fully expanded leaves of 5‐week‐old ECW20R and ECW30R (Minsavage *et al.*, 1990) pepper plants, carrying and lacking the *Bs2* gene, respectively, using a needleless syringe. The plants were kept in a growth chamber at 25 °C, *c*. 50% relative humidity, 16 h day/8 h night. HR was monitored 3 dai. For visualization of cell death, the infiltrated leaves were treated as described above for pepper leaves infiltrated with *A. citrulli* strains. Each candidate gene was tested in three independent experiments with at least three plants, with similar results being obtained among replicates and experiments.

### *Agrobacterium*‐mediated transient expression and confocal imaging

The ORFs of genes *APS58_0500*, *APS58_1448* and *APS58_4116* were amplified with specific primers (Table S7) and cloned into pEarlyGate101 binary vector (Earley *et al.*, 2006), upstream of a yellow fluorescent protein (YFP) encoding gene and an HA tag using the Gateway cloning system (ThermoFisher Scientific). The resulting plasmids were verified by sequencing and mobilized into *A. tumefaciens* GV3101 as indicated above. Transient expression experiments were performed following the protocol described by Roden *et al. *(2004b) with few modifications. Briefly, overnight cultures of *A. tumefaciens* GV3101 carrying the different plasmids were centrifuged, and pellets were resuspended in induction solution containing 10 mM MgCl_2_, 10 mM 2‐(*N*‐morpholino)‐ethanesulfonic acid (MES) and 200 mM acetosyringone (pH 5.6). The suspensions were incubated at 25 °C without shaking for 3 h. Bacterial cultures were then diluted to OD_600nm_ ~ 0.6 and infiltrated with a needleless syringe into leaves of 4‐week‐old *N. benthamiana* plants (Goodin *et al.*, 2008) that were grown in a growth chamber (16 h/26 °C in the light, 8 h/18 °C in the dark; relative humidity set to 70%). Subcellular localization of tested T3Es coupled to YFP were investigated by co‐infiltration with *A. tumefaciens* GV3101 carrying monomeric red fluorescent protein fused in frame with the ER marker HDEL (mRFP‐HDEL; Runions *et al.*, 2006; Schoberer *et al.*, 2009), the membrane‐associated SlDRP2A (L. Pizarro and M. Bar, unpublished results) fused to monomeric cherry fluorescent protein, and by staining with 1 mg/mL DAPI, which was used to detect the nucleus of the plant cells (Kapuscinski and Skoczylas, 1977). As controls, plants were infiltrated with *A. tumefaciens* GV3101 carrying pEarlyGate104 (YFP‐encoding gene). Infiltrated plants were kept in the growth chamber at similar conditions as above, and 48 hours after infiltration (hai), functional fluorophores were visualized using an SPE (Leica Microsystems, Wetzlar, Germany) or an LSM 780 (Zeiss, Oberkochen, Germany) confocal microscope. Images were acquired using two tracks: track 1 for YFP detection, exciting at 514 nm and collecting emission from the emission range 530–560 nm, and track 2 for RFP and mCherry detection, exciting at 561 nm and collecting from the emission range 588–641 nm. Images of 8 bits and 1024 × 1024 pixels were acquired using a pixel dwell time of 1.27, pixel averaging of 4 and pinhole of 1 airy unit. Analysis of colocalization was conducted with Fiji‐ImageJ using the Coloc2 tool. For calculating the Pearson correlation coefficient, 15–18 images were analysed. Signal profiles were analysed using the Plot Profile tool (Schindelin *et al.*, 2012).

## Supporting information


**Fig. S1** HrpX and HrpG are required for pathogenicity of *Acidovorax citrulli *M6. Lesions induced in a melon (cv. HA61428) leaf syringe‐infiltrated with 10^8^ cfu/mL suspensions of wild‐type M6, but not of M6 mutants defective in *hrpX* and *hrpG* genes. Partial restoration of the wild‐type phenotype was observed following transformation of the mutants with plasmids pBBR1MCS‐5::*hrpX* and pBBR1MCS‐5::*hrpG* (complementation plasmids), respectively. The picture was taken 3 days after infiltration.

**Fig. S2** Subcellular localization of APS_0500. (A) Confocal microscopy images of *Nicotiana benthamiana* epidermal cells transiently expressing APS_0500‐YFP and different endomembrane compartment markers as indicated. Representative images show APS_0500‐YFP (green), the subcellular marker: HDEL‐RFP, Free‐mCherry or SlDRP2A (magenta) and the superimposed image of both channels (merge). Pearson correlation coefficient of the co‐localization between APS_0500‐YFP and the markers (*N* = 15–18) was determined using the Coloc2 function from ImageJ. Data represented as mean ± SEM. (B) Confocal microscopy images of *N. benthamiana* epidermal cells transiently expressing the plasma membrane protein Flot1‐GFP and Free‐mCherry. All the images were acquired 48 h after *Agrobacterium tumefaciens* infiltration using Zeiss LSM780 (40×/1,2 W Korr). Scale bar 20 µm.

**Fig. S3** Subcellular localization of APS_1448. Confocal microscopy images of *Nicotiana benthamiana* epidermal cells transiently expressing APS_1448‐YFP and different endomembrane compartment markers as indicated. Representative images show APS_1448‐YFP (green), the subcellular markers HDEL‐RFP, Free‐mCherry or SlDRP2A (magenta) and the superimposed image of both channels (merge). Pearson correlation coefficient of the co‐localization between APS_1448‐YFP and the markers (*N* = 15–18) was determined using the Coloc2 function from ImageJ. Data represented as mean ± SEM. All the images were acquired 48 h after *Agrobacterium tumefaciens* infiltration using Zeiss LSM780 (40×/1,2 W Korr). Scale bar 20 µm.

**Fig. S4** Subcellular localization of APS_4116. Confocal microscopy images of *Nicotiana benthamiana* epidermal cells transiently expressing APS_1448‐YFP and different endomembrane compartment markers as indicated. Representative images show APS_1448‐YFP (green), the subcellular markers HDEL‐RFP, Free‐mCherry or SlDRP2A (magenta) and the superimposed image of both channels (merge). Pearson correlation coefficient of the co‐localization between APS_1448‐YFP and the markers (*N* = 15–18) was determined using the Coloc2 function from ImageJ. Data represented as mean ± SEM. All the images were acquired 48 h after *Agrobacterium tumefaciens* infiltration using Zeiss LSM780 (40×/1,2 W Korr). Scale bar 20 µm.

**Fig. S5** Distribution of *Acidovorax citrulli* M6 type III effectors (T3Es) according to their amino acid length. The data are from the annotation (GenBank accession CP029373.1) of the *A. citrulli* M6 ORFs**. **

**Fig. S6** Expression of effector‐AvrBs2_62‐574_::HA fusion proteins of T3Es that were tested in translocation assays. Total protein was extracted from overnight cultures of *Xanthomonas euvesicatoria* 85‐10‐hrpG*‐Δ*avrBs2* expressing CT3E‐AvrBs2_62‐574_::HA fusions in plasmid pBBR1MCS‐2*::avrBs2*_62‐574_. Proteins were analysed by western blot using HA‐tag antibody. XopS (*X. euvesicatoria* effector)‐AvrBs2_62‐574_::HA was included as positive control. Asterisks indicate the size of the expected bands.

**Table S1** Ranking and prediction scores of open reading frames of* Acidovorax citrulli* AAC00‐1 (GenBank accession CP000512.1) in the first machine‐learning run.

**Table S2** Occurrence of *Acidovorax citrulli* M6 type III effectors in other plant pathogenic *Acidovorax* species.

**Table S3** Differential gene expression as determined by RNA‐Seq between *Acidovorax citrulli* M6 and an M6 mutant strain defective in *hrpX* gene, after 72 h of growth in XVM2 minimal medium at 28 °C.

**Table S4** Perfect plant‐inducible promoter boxes in the *Acidovorax citrulli* M6 genome.

**Table S5** Ranking and prediction scores of open reading frames of *Acidovorax citrulli* M6 (GenBank accession CP029373.1) in the second machine‐learning run.

**Table S6** Bacterial strains and plasmids used in this study.

**Table S7** DNA oligonucleotide primers used in this study.

**Table S8** List and description of the features used for the first and second machine‐learning runs.

## Data Availability

The RNA‐Seq data that support the findings of this study are available at the NCBI Sequence Read Archive under BioProject PRJNA565338.
