# Supplementary figures and images for: Show me your secret(ed) weapons: a multifaceted approach reveals a wide arsenal of type III‐secreted effectors in the cucurbit pathogenic bacterium Acidovorax citrulli and novel effectors in the Acidovorax genus
Source: Mol Plant Pathol. 2019 Oct 23;21(1):17–37. doi: 10.1111/mpp.12877 (PMC6913199; doi:10.1111/mpp.12877)

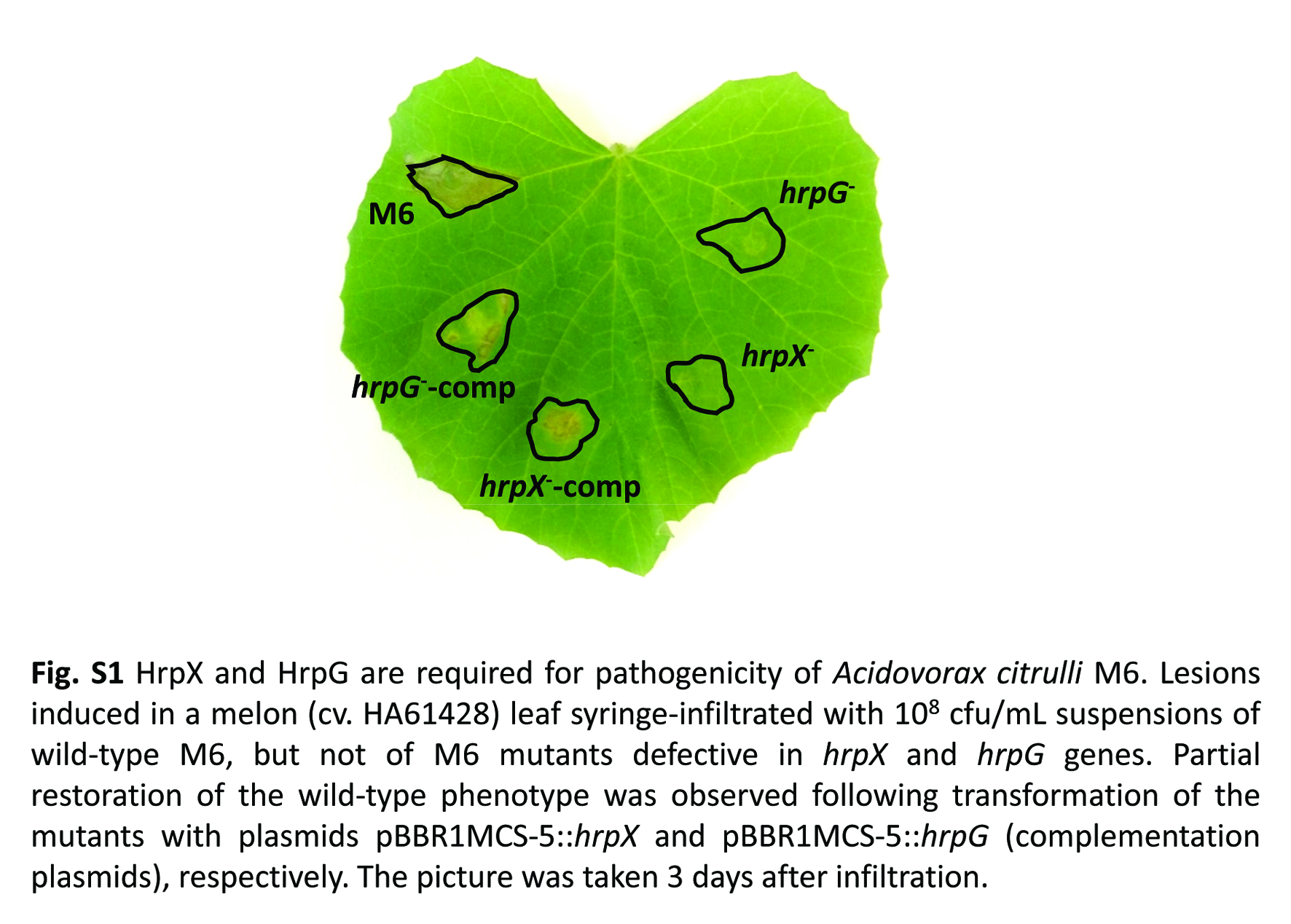

Supplement: Supplementary file 1 — Fig. S1 HrpX and HrpG are required for pathogenicity of Acidovorax citrulli M6. Lesions induced in a melon (cv. HA61428) leaf syringe‐infiltrated with 108 cfu/mL suspensions of wild‐type M6, but not of M6 mutants defective in hrpX and hrpG genes. Partial restoration of the wild‐type phenotype was observed following transformation of the mutants with plasmids pBBR1MCS‐5::hrpX and pBBR1MCS‐5::hrpG (complementation plasmids), respectively. The picture was taken 3 days after infiltration. [file MPP-21-17-s001.tif]

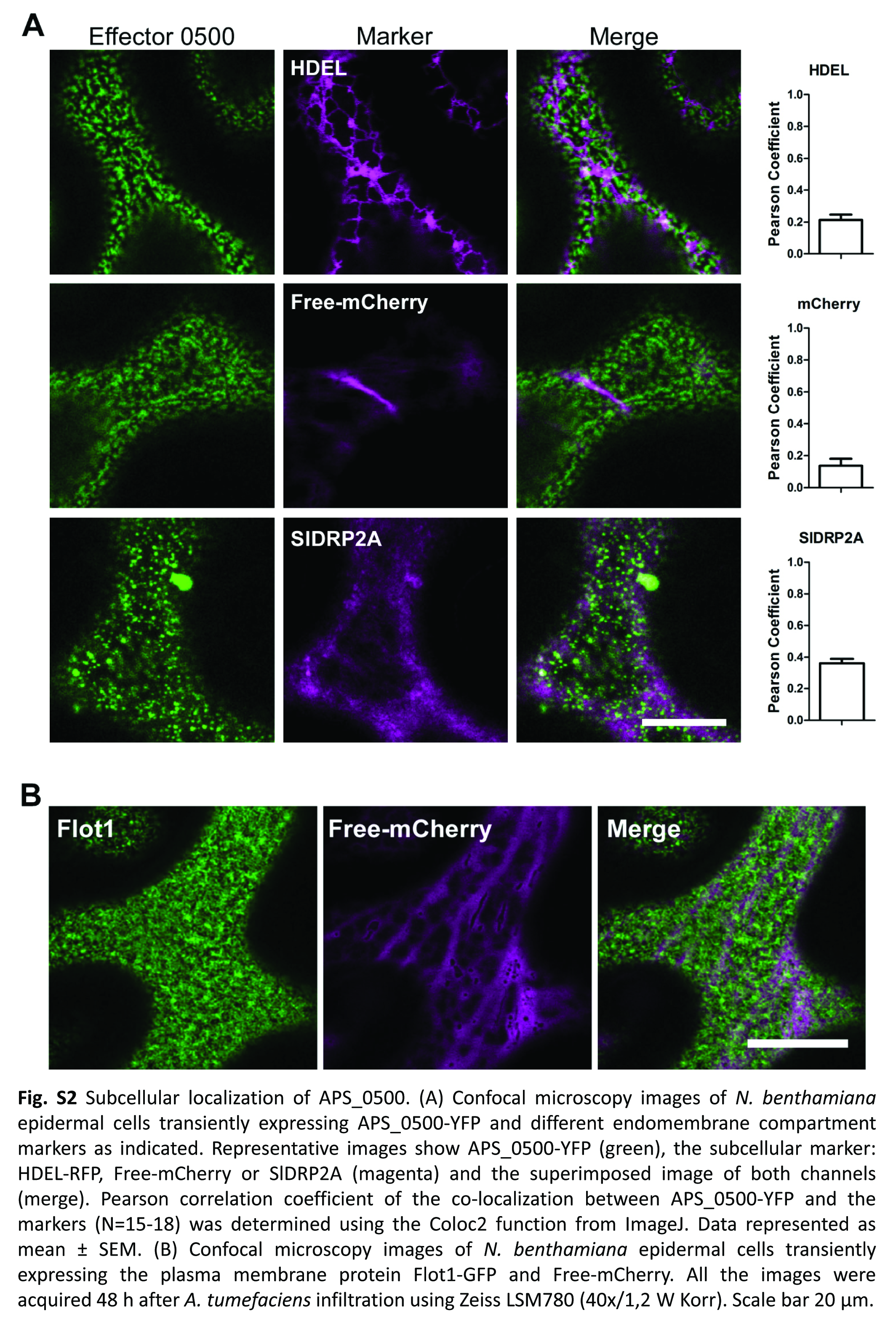

Supplement: Supplementary file 2 — Fig. S2 Subcellular localization of APS_0500. (A) Confocal microscopy images of Nicotiana benthamiana epidermal cells transiently expressing APS_0500‐YFP and different endomembrane compartment markers as indicated. Representative images show APS_0500‐YFP (green), the subcellular marker: HDEL‐RFP, Free‐mCherry or SlDRP2A (magenta) and the superimposed image of both channels (merge). Pearson correlation coefficient of the co‐localization between APS_0500‐YFP and the markers (N = 15–18) was determined using the Coloc2 function from ImageJ. Data represented as mean ± SEM. (B) Confocal microscopy images of N. benthamiana epidermal cells transiently expressing the plasma membrane protein Flot1‐GFP and Free‐mCherry. All the images were acquired 48 h after Agrobacterium tumefaciens infiltration using Zeiss LSM780 (40×/1,2 W Korr). Scale bar 20 µm. [file MPP-21-17-s002.tif]

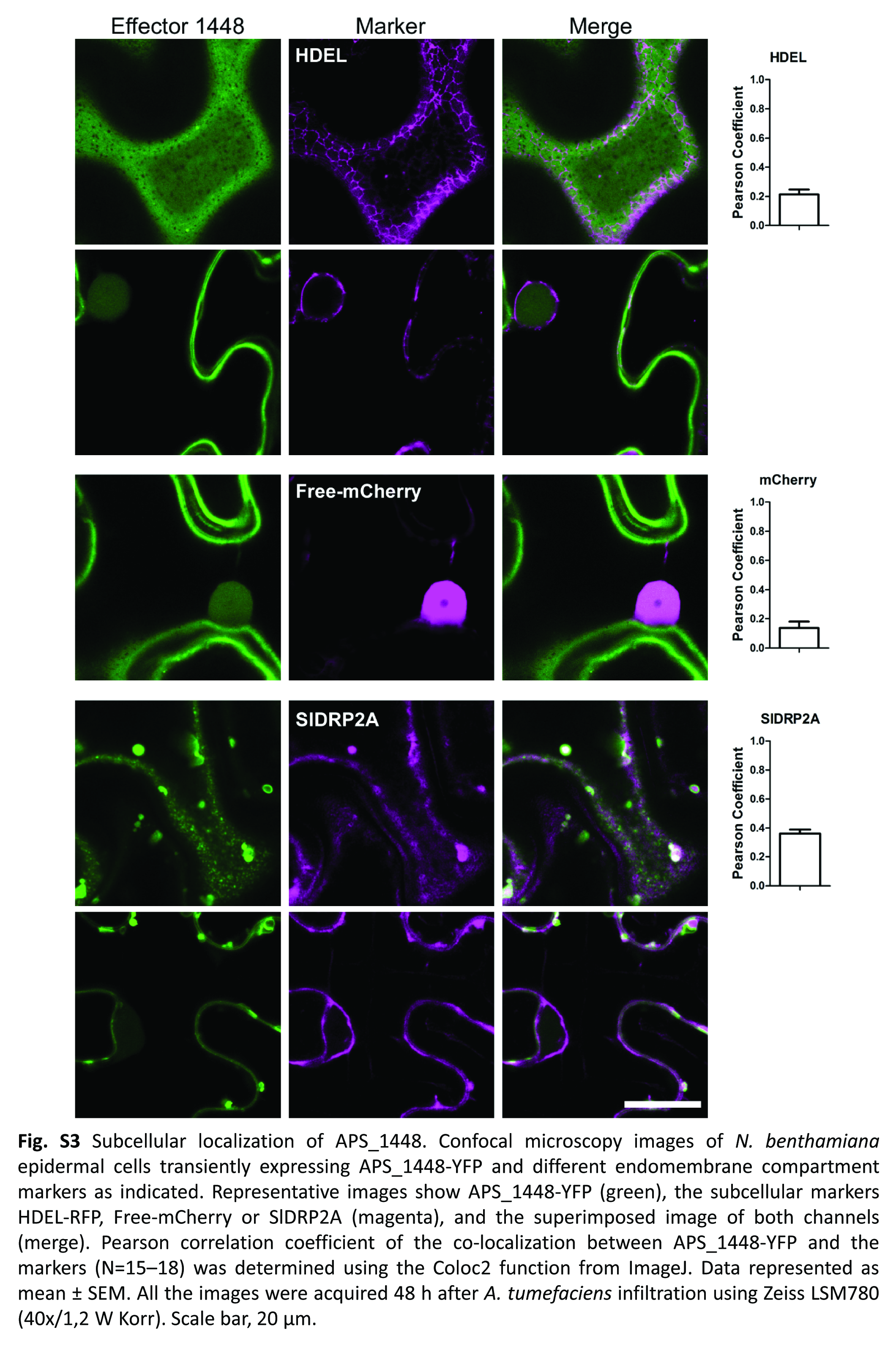

Supplement: Supplementary file 3 — Fig. S3 Subcellular localization of APS_1448. Confocal microscopy images of Nicotiana benthamiana epidermal cells transiently expressing APS_1448‐YFP and different endomembrane compartment markers as indicated. Representative images show APS_1448‐YFP (green), the subcellular markers HDEL‐RFP, Free‐mCherry or SlDRP2A (magenta) and the superimposed image of both channels (merge). Pearson correlation coefficient of the co‐localization between APS_1448‐YFP and the markers (N = 15–18) was determined using the Coloc2 function from ImageJ. Data represented as mean ± SEM. All the images were acquired 48 h after Agrobacterium tumefaciens infiltration using Zeiss LSM780 (40×/1,2 W Korr). Scale bar 20 µm. [file MPP-21-17-s003.tif]

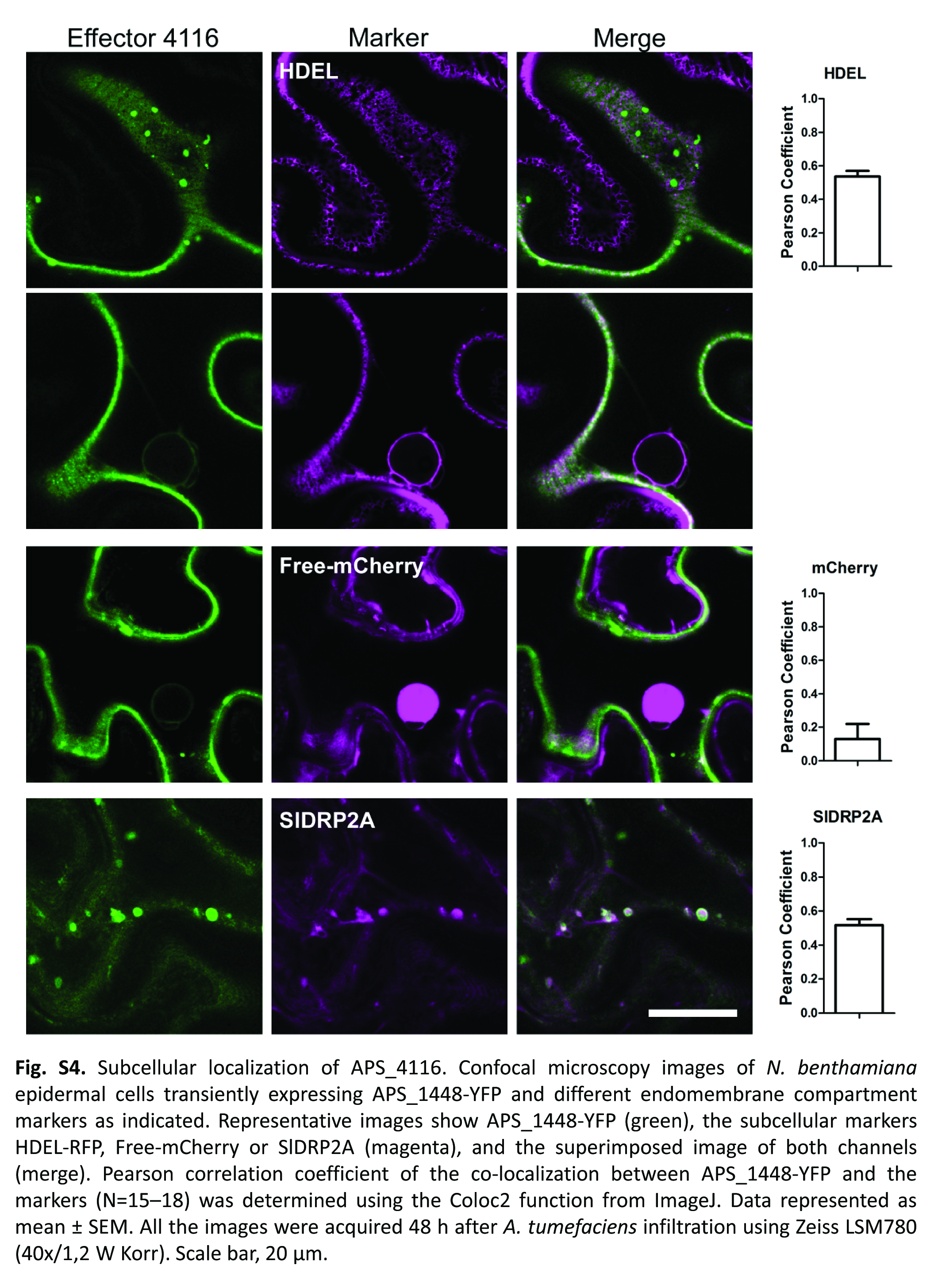

Supplement: Supplementary file 4 — Fig. S4 Subcellular localization of APS_4116. Confocal microscopy images of Nicotiana benthamiana epidermal cells transiently expressing APS_1448‐YFP and different endomembrane compartment markers as indicated. Representative images show APS_1448‐YFP (green), the subcellular markers HDEL‐RFP, Free‐mCherry or SlDRP2A (magenta) and the superimposed image of both channels (merge). Pearson correlation coefficient of the co‐localization between APS_1448‐YFP and the markers (N = 15–18) was determined using the Coloc2 function from ImageJ. Data represented as mean ± SEM. All the images were acquired 48 h after Agrobacterium tumefaciens infiltration using Zeiss LSM780 (40×/1,2 W Korr). Scale bar 20 µm. [file MPP-21-17-s004.tif]

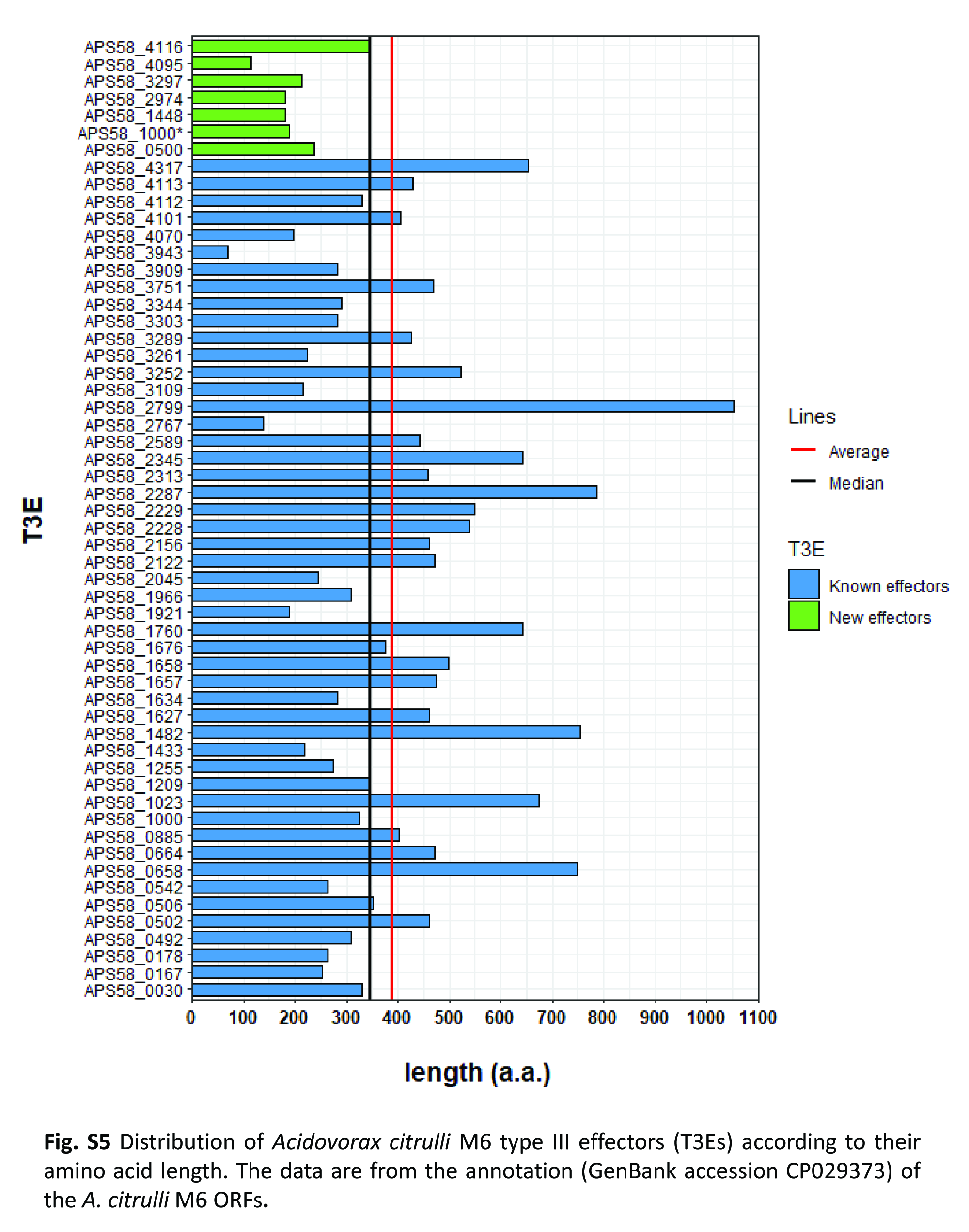

Supplement: Supplementary file 5 — Fig. S5 Distribution of Acidovorax citrulli M6 type III effectors (T3Es) according to their amino acid length. The data are from the annotation (GenBank accession CP029373.1) of the A. citrulli M6 ORFs. [file MPP-21-17-s005.tif]

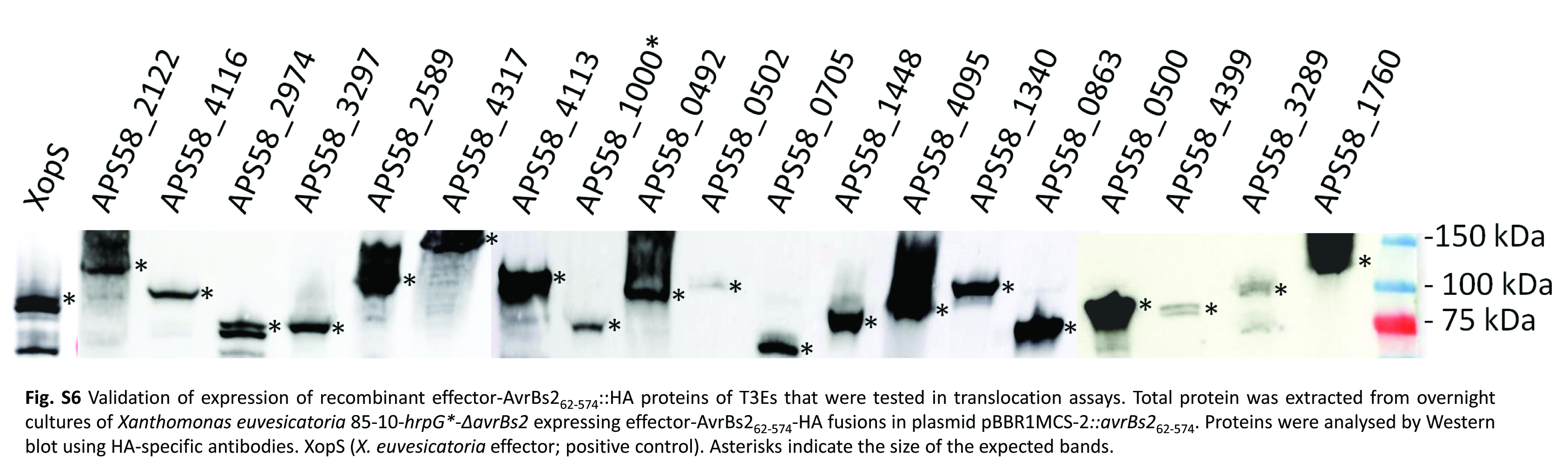

Supplement: Supplementary file 6 — Fig. S6 Expression of effector‐AvrBs262‐574::HA fusion proteins of T3Es that were tested in translocation assays. Total protein was extracted from overnight cultures of Xanthomonas euvesicatoria 85‐10‐hrpG*‐ΔavrBs2 expressing CT3E‐AvrBs262‐574::HA fusions in plasmid pBBR1MCS‐2::avrBs2 62‐574. Proteins were analysed by western blot using HA‐tag antibody. XopS (X. euvesicatoria effector)‐AvrBs262‐574::HA was included as positive control. Asterisks indicate the size of the expected bands. [file MPP-21-17-s006.tif]
