# Supplementary material for: Show me your secret(ed) weapons: a multifaceted approach reveals a wide arsenal of type III‐secreted effectors in the cucurbit pathogenic bacterium Acidovorax citrulli and novel effectors in the Acidovorax genus
Source: Mol Plant Pathol. 2019 Oct 23;21(1):17–37. doi: 10.1111/mpp.12877 (PMC6913199; doi:10.1111/mpp.12877)
Supplement: Supplementary file 12 — Table S6 Bacterial strains and plasmids used in this study. [file MPP-21-17-s012.docx]

**Table S6** Bacterial strains and plasmids used in this study.

| Strain/plasmid | Relevant properties | Source or reference |
| --- | --- | --- |
| ***Acidovorax citrulli*** | | |
| M6 | wild type, group I strain; Ap^R^ | Burdman *et al.*, 2005 |
| M6 *hrcV^-^* | M6 derivate disrupted in the *hrcV* gene; Ap^R^, Km^R^ | Bahar and Burdman, 2010 |
| M6 *hrpX^-^* | M6 derivate disrupted in the *hrpX* gene; Ap^R^, Km^R^ | This study |
| M6 *hrpG^-^* | M6 derivate disrupted in the *hrpG* gene; Ap^R^, Km^R^ | This study |
| M6 *hrpX^-^* (pBBR1MCS-5*::hrpX*) | M6 *hrpX*^-^ mutant complemented with pBBR1MCS-5*::hrpX*; Ap^R^, Km^R^ Gm^R^ | This study |
| M6 *hrpG^-^* (pBBR1MCS-5*::hrpG*) | M6 *hrpG*^-^ mutant complemented with pBBR1MCS-5*::hrpG*; Ap^R^, Km^R^ Gm^R^ | This study |
| ***Xanthomonas euvesicatoria*** | | |
| 85-10 *hrpG**∆*avrBs2* | 85-10 derivative containing the *hrpG** mutation (overexpressing this gene) and a deletion in *avrBs2*; Rif^R^, Gm^R^ | Roden *et al.*, 2004 |
| 85-10 *hrpG**∆*hrpF* | 85-10 derivative containing the *hrpG** mutation and a deletion in *hrpF*; Rif^R^ | Casper-Lindley *et al.*, 2002 |
| 85-10 *hrpG**∆*avrBs2* (pBBR1MCS-2::*avrBs2_62-574_*) | 85-10 *hrpG**∆*avrBs2* derivate carrying a plasmid with the AvrBs2 HR domain without the N-terminal translocation signal (AvrBs2_62-574_); negative control in translocation assays; Rif^R^, Km^R^ | Teper *et al.*, 2015 |
| 85-10 *hrpG**∆*avrBs2* (pBBR1MCS-2*::xopS::avrBs2_62-574_*) | 85-10 *hrpG**∆*avrBs2* derivate carrying a plasmid with the *X. euvesicatoria* 85-10 *xopS* ORF without the stop codon fused in frame with AvrBs2_62-574_; positive control in translocation assays; Rif^R^, Km^R^ | Teper *et al.*, 2015 |
| 85-10 *hrpG**∆*avrBs2* (pBBR1MCS-2*::APS58_XXXX::avrBs2_62-574_*) | Nineteen 85-10 *hrpG**∆*avrBs2* derivates carrying a plasmid with an *APS58_XXXX* ORF without the stop codon fused in frame with AvrBs2_62-574_; *XXXX* refers to the gene ID of *A. citrulli* M6 genes tested in translocation assays; Rif^R^, Km^R^ | This study |
| 85-10 *hrpG**∆*hrpF* (pBBR1MCS-2*::APS58_XXXX::avrBs2_62-574_*) | Nineteen 85-10 *hrpG**∆*hrpF* derivates carrying a plasmid with an *APS58_XXXX* ORFs without the stop codon fused in frame with AvrBs2_62-574_; *XXXX* refers to the gene ID of *A. citrulli* M6 genes tested in translocation assays; Rif^R^, Km^R^ | This study |
| ***Agrobacterium tumefaciens*** | | |
| GV3101 | wild type; Rif^R^ | Rotino and Gleddie, 1990 |
| ***Escherichia coli*** | | |
| DB3.1 | *gyrA462*, *endA1*, ∆(*sr1-recA*), *mcrB*, *mrr*, *hsdS20*, *glnV44* (=*supE44*), *ara14*, *galK2*, *lacY1*, *proA2*, *rpsL20*, *xyl5*, *leuB6*, *mtl1* | Invitrogen (Carlsbad, California) |
| DH5α | *supE44*, ∆*lacU169*, *hsdR17*, *recA1*, *endA1*, *gyrA96*, *thi-1*, *relA1*, Nx^R^ | Sambrook *et al.*, 1989 |
| S17-1 λpir | ∆lysogenic S17-1 derivate producing π protein for replication of plasmids carrying *oriR6K*; *recA*, *pro*, *hsdR,* RP4-2-Tc::Mu-Km::Tn7, λ-pir | Simon *et al.*, 1983 |
| **Plasmids** | | |
| pJP5603 | R6K-based suicide vector; requires the pir-encoded π protein for replication; used for mutagenesis of *A. citrulli*; Km^R^ | Penfold and Pemberton, 1992 |
| pBBR1MCS-5 | P_T7_rep broad host expression vector; Gm^R^ | Kovach *et al.*, 1995 |
| pJP5603::*hrpX*_int_ | pJP5603 carrying an internal fragment (383 bp) of *hrpX* with an early stop codon, inserted into the *Bam*HI/*Eco*RI sites; used for generation of *A. citrulli* M6 *hrpX*^-^ mutant by insertional mutagenesis following single homologous recombination; Km^R^ | This study |
| pBBR1MCS-5::*hrpX* | pBBR1MCS-5 carrying the *hrpX* ORF (1407 bp) inserted into the *Eco*RI/*Bam*HI sites and under the control of the lacZα promoter; used for complementation of the *A. citrulli* M6 *hrpX*^-^ mutant; Gm^R^ | This study |
| pJP5603*::hrpG*_int_ | pJP5603 carrying an internal fragment (438 bp) of *hrpG* with an early stop codon, inserted into the *Bam*HI/*Eco*RI sites; used for generation of *A. citrulli* M6 *hrpG*^-^ mutant by insertional mutagenesis following single homologous recombination; Km^R^ | This study |
| pBBR1MCS-5*::hrpG* | pBBR1MCS-5 carrying the *hrpG* ORF (801 bp) inserted into the *Eco*RI/*Bam*HI sites and under the control of the lacZα promoter; used for complementation of the *A. citrulli* M6 *hrpG*^-^ mutant; Gm^R^ | This study |
| pDONR207 | Gateway donor vector; Gm^R^ | Invitrogen |
| pEarleyGate100 | Gateway-compatible plant transformation vector; Km^R^ | Earley *et al.*, 2006 |
| pEarleyGate101 | Gateway-compatible plant transformation vector with YFP and HA C-terminal tags; Km^R^ | Earley *et al.,* 2006 |
| pEarleyGate104 | Gateway-compatible plant transformation vector with a YFP N-terminal tag; Km^R^ | Earley *et al.*, 2006 |
| pDONR207*::APS58_0500* | pDONR207 carrying the *APS58_0500* ORF without the stop codon (711 bp); Gm^R^ | This study |
| pEarleyGate101*::APS58_0500* | pEarleyGate101 carrying the *APS58_0500* ORF without the stop codon fused to YFP and HA; Km^R^ | This study |
| pDONR207*::APS58_1448* | pDONR207 carrying the *APS58_1448* ORF without the stop codon (543 bp); Gm^R^ | This study |
| pEarleyGate101*::APS58_1448* | pEarleyGate101 carrying the *APS58_1448* ORF without the stop codon fused to YFP and HA; Km^R^ | This study |
| pDONR207*::APS58_4116* | pDONR207 carrying the *APS58_4116* ORF without the stop codon (1029 bp); Gm^R^ | This study |
| pEarleyGate101*::APS58_4116* | pEarleyGate101 carrying the *APS58_4116* ORF without the stop codon fused to YFP and HA; Km^R^ | This study |
| pmRFP-HDEL | Plasmid used for transient expression of the endoplasmic reticulum marker HDEL fused to red fluorescent protein (RFP); Km^R^ | Runions *et al*., 2006; Schoberer *et al*., 2009 |
| pBIN20::ER‐rk (CD3‐959) | pBIN20 carrying HDEL endoplasmic reticulum retention peptide fused to mCherry; Km^R^ | Nelson *et al.*, 2007 |
| pCAMBIA1200::GFP-Flot1 | pCAMBIA1200 carrying the plasma membrane protein Flot1 from *Arabidopsis thaliana* fused to green fluorescent protein (GFP) under the CaMV 35S promoter; Km^R^ | Li *et al.*, 2012 |
| pBINPLUS::SlDRP2A-mCherry | pBINPLUS carrying the Dynamin SlDRP2A from *Solanum lycopersicum* fused to mCherry under the CaMV 35S promoter; Km^R^ | L. Pizarro and M. Bar, unpublished |
| pBINPLUS::mCherry | pBINPLUS carrying mCherry under the CaMV 35S promoter; Km^R^ | Leibman-Markus *et al.*, 2018. |
| pBBR1MCS-2*::avrBs2_62-574_* | pBBR1MCS-2 carrying the region encoding the HR-inducing domain of AvrBs2, AvrBs2_62-574_, inserted into the *Xba*I/*Sac*I sites and upstream of an HA tag; Km^R^ | Teper *et al.*, 2015 |
| pBBR1MCS-2*::APS58_XXXX::avrBs2_62-574_* | pBBR1MCS-2*::avrBs2_62-574_* carrying *APS58_XXXX* ORFs without the stop codon, inserted into the *Sal*I/*Xba*I sites upstream and in frame with *avrBs2_62-574_*; used to assess translocation; seventeen constructs with *XXXX* being *0492*, *0502*, *0705*, *0863*, *1000** (non-annotated gene; located between genes *APS58_0999* and *APS58_1000*), *1340*, *1448*, *2122*, *2589*, *2974*, *3289*, *3297*, *4095*, *4113*, *4116*, *4317*, *4399*; Km^R^ | This study |
| pBBR1MCS-2*::APS58_XXXX::avrBs2_62-574_* | pBBR1MCS-2*::avrBs2_62-574_* carrying *APS58_XXXX* ORFs without the stop codon, inserted into the *Xho*I/*Xba*I sites upstream and in frame with *avrBs2_62-574_*; used to assess translocation; two constructs with *XXXX* being *0500* and *1760*; Km^R^ | This study |

^*^ Ap^R^, Gm^R^, Km^R^ and Rif^R^ indicate resistance to ampicillin, gentamicin, kanamycin and rifampicin, respectively.

**References**

**Bahar, O. and Burdman, S.** (2010) Bacterial fruit blotch: a threat to the cucurbit industry. *Israel J. Plant Sci.* **58,** 19-31.

**Burdman, S., Kots, N., Kritzman, G. and Kopelowitz, J.** (2005) Molecular, physiological, and host-range characterization of *Acidovorax avenae* subsp. *citrulli* isolates from watermelon and melon in Israel. *Plant Dis*. **89,** 1339-1347.

**Casper-Lindley, C., Dahlbeck, D., Clark, E.T., and Staskawicz, B.J.** (2002) Direct biochemical evidence for type III secretion-dependent translocation of the AvrBs2 effector protein into plant cells. *Proc. Natl. Acad. Sci. USA,* **99,** 8336-8341.

**Earley, K.W., Haag, J.R., Pontes, O., Opper, K., Juehne, T., Song, K. and Pikaard, C.S.** (2006) Gateway-compatible vectors for plant functional genomics and proteomics. *Plant J*. **45,** 616-629.

**Kovach, M.E., Elzer, P.H., Hill, D.S., Robertson, G.T., Farris, M.A., Roop, R.M. and Peterson, K.M.** (1995) Four new derivatives of the broad-host-range cloning vector pBBR1MCS, carrying different antibiotic-resistance cassettes. *Gene,* **166,** 175-176.

**Leibman-Markus, M., Pizarro, L., Schuster, S., Lin, Z.J.D., Gershony, O., Bar, M., Coaker, G. and Avni, A.** (2018) The intracellular nucleotide-binding leucine-rich repeat receptor (SlNRC4a) enhances immune signalling elicited by extracellular perception. *Plant Cell Environ.* **41,** 2313-2327.

**Li, R., Liu, P., Wan, Y., Chen, T., Wang, Q., Mettbach, U., Baluska, F., Samaj, J., Fang, X., Lucas, W.J. and Lin, J.** (2012) A membrane microdomain-associated protein, Arabidopsis Flot1, is involved in a clathrin-independent endocytic pathway and is required for seedling development. *Plant Cell*, **24,** 2105-2122.

**Nelson, B.K., Cai, X. and Nebenfuhr, A.** (2007) A multicolored set of in vivo organelle markers for co-localization studies in Arabidopsis and other plants. *Plant J.* **51,** 1126-1136.

**Penfold, R.J. and Pemberton, J.M.** (1992) An improved suicide vector for construction of chromosomal insertion mutations in bacteria. *Gene,* **118,** 145-146.

**Roden, J.A., Belt, B., Ross, J.B., Tachibana, T., Vargas, J. and Mudgett, M.B.** (2004a) A genetic screen to isolate type III effectors translocated into pepper cells during *Xanthomonas* infection. *Proc. Natl. Acad. Sci. USA,* **101,** 16624-16629.

**Rotino, G.L. and Gleddie, S.** (1990) Transformation of eggplant (*Solanum melongena* L.) using a binary *Agrobacterium tumefaciens* vector. *Plant Cell Rep.* **9,** 26-29.

**Runions, J., Brach, T., Kühner, S. and Hawes, C.** (2006) Photoactivation of GFP reveals protein dynamics within the endoplasmic reticulum membrane. *J. Exp. Bot.* **57,** 43-50.

**Sambrook, J., Fritsch, E.F. and Maniatis T.** (1989) Molecular Cloning: A Laboratory Manual. Cold Spring Harbor, NY: Cold Spring Harbor Laboratory.

**Schoberer, J., Vavra, U., Stadlmann, J., Hawes, C., Mach, L., Steinkellner, H. and Strasser, R.** (2009) Arginine/lysine residues in the cytoplasmic tail promote ER export of plant glycosylation enzymes. *Traffic,* **10,** 101-115.

**Simon, R., Priefer, U. and Puhler, A.** (1983) A broad host range mobilization system for in vivo genetic engineering: Transposon mutagenesis in gram negative bacteria. *Biotechnology,* **1,** 784-791.

**Teper, D., Burstein, D., Salomon, D., Gershovitz, M., Pupko, T. and Sessa, G.** (2016) Identification of novel *Xanthomonas euvesicatoria* type III effector proteins by a machine-learning approach. *Mol. Plant Pathol*. **17,** 398-411.
